# Radiomics Nomogram for Prediction of Peritoneal Metastasis in Patients With Gastric Cancer

**DOI:** 10.3389/fonc.2020.01416

**Published:** 2020-08-20

**Authors:** Weicai Huang, Kangneng Zhou, Yuming Jiang, Chuanli Chen, Qingyu Yuan, Zhen Han, Jingjing Xie, Shitong Yu, Zepang Sun, Yanfeng Hu, Jiang Yu, Hao Liu, Ruoxiu Xiao, Yikai Xu, Zhiwei Zhou, Guoxin Li

**Affiliations:** ^1^Department of General Surgery, Nanfang Hospital, Southern Medical University, Guangzhou, China; ^2^School of Computer and Communication Engineering, University of Science and Technology Beijing, Beijing, China; ^3^Department of Medical Imaging Center, Nanfang Hospital, Southern Medical University, Guangzhou, China; ^4^Center for Drug and Clinical Research, Nanfang Hospital, Southern Medical University, Guangzhou, China; ^5^Department of Gastric Surgery, Sun Yat-sen University Cancer Center, Guangzhou, China; ^6^State Key Laboratory of Oncology in South China, Collaborative Innovation Center for Cancer Medicine, Guangzhou, China

**Keywords:** gastric cancer, peritoneum, metastasis, radiomics, nomogram

## Abstract

**Objective:** The aim of this study is to evaluate whether radiomics imaging signatures based on computed tomography (CT) could predict peritoneal metastasis (PM) in gastric cancer (GC) and to develop a nomogram for preoperative prediction of PM status.

**Methods:** We collected CT images of pathological T4 gastric cancer in 955 consecutive patients of two cancer centers to analyze the radiomics features retrospectively and then developed and validated the prediction model built from 292 quantitative image features in the training cohort and two validation cohorts. Lasso regression model was applied for selecting feature and constructing radiomics signature. Predicting model was developed by multivariable logistic regression analysis. Radiomics nomogram was developed by the incorporation of radiomics signature and clinical *T* and *N* stage. Calibration, discrimination, and clinical usefulness were used to evaluate the performance of the nomogram.

**Results:** In training and validation cohorts, PM status was associated with the radiomics signature significantly. It was found that the radiomics signature was an independent predictor for peritoneal metastasis in multivariable logistic analysis. For training and internal and external validation cohorts, the area under the receiver operating characteristic curves (AUCs) of radiomics signature for predicting PM were 0.751 (95%CI, 0.703–0.799), 0.802 (95%CI, 0.691–0.912), and 0.745 (95%CI, 0.683–0.806), respectively. Furthermore, for training and internal and external validation cohorts, the AUCs of radiomics nomogram for predicting PM were 0.792 (95%CI, 0.748–0.836), 0.870 (95%CI, 0.795–0.946), and 0.815 (95%CI, 0.763–0.867), respectively.

**Conclusions:** CT-based radiomics signature could predict peritoneal metastasis, and the radiomics nomogram can make a meaningful contribution for predicting PM status in GC patient preoperatively.

## Introduction

Gastric cancer (GC) is one of the most common human malignancies and the third leading cause of cancer-related deaths worldwide (1–3). Surgical resection is the major treatment for GC patient (4); however, patients in advanced gastric cancer with peritoneal metastasis, a non-curable factor, showed poor prognosis (5). Peritoneal metastasis (PM) primary occurs in T4 stage (6, 7). Accurate evaluation of PM status in GC patients is essential for treatment decision and prognosis. It was found that some biomarkers and histopathological factors (e.g., *T, N* staging, Greater Omental Milky Spot, and Troponin I2) could predict PM status in GC (7, 8), but they just provided low prediction or were merely available postoperatively. Preoperative assessment of PM can provide useful information for performing adjuvant treatment and avoid unnecessary surgical resection, thus contributing to pretreatment decision. Computed tomography (CT), which could detect obvious parietal peritoneum thickening and ascites, is regarded as a popular non-invasive method to diagnose PM (9). However, the signs of obvious parietal peritoneum thickening or large amount of ascites did not always exist in every GC patient with PM. There still exist GC patients who were CT-diagnosed PM-negative but confirmed PM-positive during subsequent laparoscopies (9). Therefore, CT-diagnosed PM has low sensitivity. Laparoscopy, a golden criterion for detecting PM status, is strongly recommended by the European Society for Medical Oncology (ESMO) and National Comprehensive Cancer Network (NCCN) guidelines to perform for its diagnosis but is controversial due to the different clinical *T* stage and health status; for example, some patients could not suffer the intraperitoneal high pressure during the performance of laparoscopy. Laparoscopy is an invasive diagnostic procedure; hence, it is not suitable for each patient. Therefore, accurate preoperative prediction of PM status is very important for GC patients, especially at the late stage.

Radiomics, an arising field that involves converting digital medical images into mineable data, analyzing data, and improving medical decision, has attracted increasing attention in recent years (10, 11). With radiomics, the accuracy of diagnosis, prognosis, and prediction could be improved, especially in oncology (12). Radiomics enables the non-invasive profiling of tumor heterogeneity (13, 14) through integrating complex imaging features. The applications of radiomics mainly focus on individualized therapy associated with cancer such as tumor detection, lymph node metastasis (LNM), subtype classification, survival, and treatment reaction assessment (13, 15–17). It was reported that CT texture was associated with prognosis in patients with GC (18); however, an ideal method that can change complex imaging features into a signature for predicting PM is still urgent to be developed. For GC patients especially those in clinical T3–T4, who are more likely to have higher risk of PM, developing a predictive model with radiomics signature to predict PM status is quite necessary.

The purpose of this study was to establish a radiomics signature for predicting the PM status in GC patients on the basis of preoperative CT information and to further develop a radiomics model that incorporates the clinicopathological findings and radiomics signature for the personal prediction of PM status in patients with GC preoperatively.

## Materials and Methods

### Patients

This study was approved by the ethics committee of every participating center, and the informed consent requirement was waved. We retrospectively selected three independent cohorts of patients with GC in pathological T4 stage. The training and internal validation cohorts comprising 562 and 106 consecutive patients, respectively, with total or partial radical gastrectomy were obtained from Sun Yat-sen University Cancer Center between January 2008 and December 2012 and January 2013 and December 2013, respectively. The external validation cohort that comprised 287 consecutive patients was obtained from Nanfang Hospital of Southern Medical University (Guangzhou, China) between January 2007 and December 2013. Clinicopathological data of each patient were collected retrospectively. Table 1 shows the characteristics of the 955 GC patients. Patients were included if they met the following criteria: performed standard unenhanced and contrast-enhanced abdominal CT <4 weeks before surgery, with fundamental clinicopathological data, without combined malignant tumor, without preoperative chemotherapy, and being confirmed T4 GC histologically. Exclusion criteria were as follows: CT could not distinguish the lesions of the neoplasm, and anticancer therapy was performed previously. Fundamental clinicopathological data, such as gender, age, size, location, cancer antigen 19-9 (CA19-9), carcinoembryonic antigen (CEA), status of preoperative differentiation, were obtained from medical records. We also collected the dates of baseline CT imaging and the clinical *T* stage (cT) and *N* stage (cN) of patients. The details are shown in Figure S1.

**Table 1:** Characteristics of patients with gastric cancer (GC) in each cohort.

**Variables**	**Training Cohort (*****N*** **= 562)**		**Internal Validation Cohort (*****N*** **= 106)**		**External Validation Cohort (*****N*** **= 287)**	
	***N***	**%**	***N***	**%**	***N***	**%**
**AGE (YEARS)**						
≥60	238	42.3	46	43.4	125	43.6
<60	324	57.7	60	56.6	162	56.4
**GENDER**						
Male	385	68.5	76	71.7	207	72.1
Female	177	31.5	30	28.3	80	27.9
**SIZE**						
≥4 cm	409	72.8	79	74.5	196	68.3
<4 cm	153	27.2	27	25.5	91	31.7
**DIFFERENTIATION**						
Well or moderate	75	13.3	21	19.8	60	20.9
Poor or undifferentiation	487	86.7	85	80.2	227	79.1
**LAUREN TYPE**						
Intestinal	157	27.9	36	34	100	34.8
Mixed and diffuse	405	72.1	70	66	187	65.2
**LOCATION**						
Cardia	218	38.8	32	30.2	59	20.6
Body	113	20.1	31	29.2	48	16.7
Antrum	183	32.6	36	34	140	48.8
Whole	48	8.5	7	6.6	40	13.9
**CEA**						
Elevated	139	24.7	30	28.3	50	17.4
Normal	423	75.3	76	71.7	237	82.6
**CA19-9**						
Elevated	130	23.1	19	17.9	62	21.6
Normal	432	76.9	87	82.1	225	78.4
**cT STAGE**						
T3	99	17.6	27	25.5	34	11.8
T4a	337	60	48	45.3	232	80.8
T4b	126	22.4	31	29.2	21	7.4
**cN STAGE**						
N0	159	28.3	23	21.7	69	24
N1	131	23.3	18	17	58	20.2
N2	125	22.2	22	20.8	69	24
N3	147	26.2	43	40.6	91	31.8
**PM**						
PM(–)	472	84	89	84	225	78.4
PM(+)	90	16	17	16	62	21.6

Peritoneal metastasis status was divided into two outcome categories: PM-negative status [PM(–)] and PM-positive status [PM(+)]. All the diagnoses of PM status were based on the laparoscopy surgery and pathological examination.

### Image Acquisition

Contrast-enhanced abdominal CT of patients was performed by the multidetector row CT (MDCT) systems (256-MDCT scanner Brilliance iCT, Philips Healthcare, Cleveland, OH, USA; 64-section LightSpeed VCT, GE Medical Systems, Milwaukee, WI, USA; or GE Lightspeed 16, GE Healthcare Milwaukee, WI, USA). To standardize the image acquisition, portal venous phase contrast-enhanced CT images were retrieved from the picture archiving and communication system (PACS) (Carestream, Canada) in Digital Imaging and Communications in Medicine (DICOM) format for feature extraction (19). The details are shown in Supplementary Materials.

### Imaging Texture Analysis

All the CT images were reviewed by two experienced radiologists who both had clinical experience in abdominal CT study interpretation for more than 10 years. Based on the consensus of these two radiologists, the tumor manual segmentation was performed and checked with *ITK-SNAP* software (www.itksnap.org) (20). They constructed manually a single region of interest (ROI) that covered the entire area of the lesion on the transverse image section, which depicted the maximum lesion diameter for each lesion. Based on the above procedure, we used an available radiomics analysis package (https://github.com/mvallieres/radiomics/) in Matlab R2016a (The MathWorks Inc.) to extract and calculated the radiomics features of these images. The inter- and intraobserver variability of radiomics feature extraction of the two radiologists was initially analyzed with 100 randomly chosen images for ROI-based texture feature extraction; details are shown in Supplementary Methods 2. The final feature pool included first-order intensity features, shape features, and second- and higher-order textural features. The detailed mathematical definitions of all imaging features are listed in Supplementary Materials.

### Radiomics Feature Selection and Signature Development

In order to predict the PM status of patients with GC, we used the least absolute shrinkage and selection operator (LASSO) logistic regression model to select the optimal radiomics features from the primary texture features, and then, the development of the radiomics score (Rad-score) was constructed in the training cohort (21). For further detecting and addressing the collinearity among features, scatterplot correlation matrix with Person correlation coefficient was applied to investigate the interrelationship among the primary selected features and PM status, and if features had a correlation coefficient that was higher than 0.80 between each other, then the one with the highest collinearity was excluded from the analysis (22–24). In this study, we used the R software (version 3.5.3) with the “glmnet” package to perform the LASSO regression (25, 26). A detailed information is provided in Supplementary Materials.

### Development of an Individualized Prediction Model

Estimation of univariate relationships between PM status and potential predictors were developed with logistic regression analysis. The basis of the PM status prediction model was established by multivariate logistic regression analysis. Different variables were analyzed by using univariate logistic regression analysis in the training cohort, and statistically significant variable values with *P* < 0.05 in each cohort were then entered into the multivariate analyses. We used the likelihood ratio test with Akaike's information criterion as the stopping rule to apply the backward step-wise selection (27). Based on the prediction model, a nomogram was then constructed.

### Validation of the Prediction Model

The accuracy of the prediction model was assessed by measuring both discrimination and calibration. We used 1,000 bootstrapping resamples for evaluating both discrimination and calibration. Discriminative ability was measured by the area under the receiver operating characteristic curve (AUC). The consistency between the predicted and actual probability of PM status was graphically represented by calibration plots.

### Clinical Use

The potential net benefit of the predictive models was assessed by the decision curve analysis (DCA), which is popular as a new method for evaluating predictive model recently (28). The decision strategy based on every threshold probability would show a potential net benefit by using this method. In this study, we quantified the net benefits at different threshold probabilities with the use of the DCA to identify the clinical usefulness of the Rad-score, cT stage, cN stage, and radiomics nomogram.

### Statistical Analysis

For continuous variable values, the two-tailed *t-*test, unpaired, one-way ANOVA, or Mann–Whitney test were used for comparison. For categorical variables, the χ^2^ test or Fisher's exact test was used to compare. Standardization of the image features was applied by transforming the data of each feature into new scores with a mean of zero and a standard deviation of 1 (z-score transformation) (29, 30). Most of the statistical tests were examined by using SPSS version 21.0 (IBM) and R version 3.5.3 (http://www.r-project.org), and the nomograms and calibration plots were conducted by using the R version 3.5.3 with rms package. For all tests, *P* < 0.05 was thought to be statistically significant.

## Results

### Clinical Characteristics

Table 1, Tables S1–S3 are the clinicopathological characteristics of the training cohort (*n* = 562), internal (*n* = 106), and external validation cohort (*n* = 287). As are shown in the tables, the clinical characteristics of patients among the three cohorts have no statistical difference. PM(+) status occurred in 90 (16.0%) patients, 17 (16.0%) patients, and 62 (21.6%) patients in the training, internal, and external validation cohort, respectively (Table 1).

The inter- and intraclass correlation coefficients (ICCs) for the two radiologists' extracted features were both higher than 0.75, indicating that the inter- and intraobserver agreements of radiomics feature extraction of the two radiologists were good, so all outcomes were calculated on the basis of the measurements of the first radiologist. Details are summarized in Supplementary Materials.

### Feature Selection and Radiomics Signature Building

On the basis of 562 patients in the primary cohort, 292 features were extracted from the CT image by using Matlab R2016a. Then, the 292 features were reduced to 11 potential predictors (Figure S2) by being featured with non-zero coefficients in the LASSO logistic regression model. Features that showed high collinearity with each other were excluded from analysis (Figure S3). Finally, four PM status-related features were selected for constructing a meaningful radiomics signature, presented with a Rad-score calculation formula: **Rad-score** = −1.429359e−03 × Eccentricity + 1.232216e−02 × Exten – 9.887834e−02 × GLCM_IMC1-0.5 + 8.977322e-02 × GLCM_MaximumProbability-0.5. Details are shown in Supplementary Materials. The associations among the clinicopathological characteristics, Rad-score, and PM status in different cohorts are showed in Tables S1–S3.

The relationships among PM status, Rad-score, and clinical characteristics in each cohort were determined by three heatmaps (Figures S4–S6). In the training and internal and external validation cohorts, significant positive relationship was found between Rad-score and PM status.

### Diagnostic Validation of Radiomics Signature

**Figure 1:**
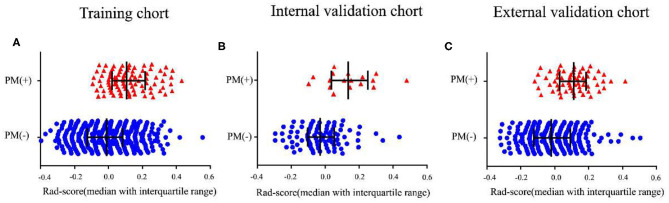
Distribution of radiomics scores according to the PM status. **(A–C)** The values of the radiomics scores (Rad-scores) of each patient and the median values with interquartile range of Rad-scores. PM, peritoneal metastasis status.

In the training cohort, the Rad-score between PM(–) and PM(+) patients was significantly different (*P* < 0.01, Figure 1). Similarly, in the two validation cohorts, the Rad-score was also confirmed to be significantly different between PM(–) and PM(+) patients (*P* < 0.01, Figure 1). Higher Rad-scores were found in PM(+) patients in the training and internal and external validation cohorts consistently. The radiomics signature displayed an AUC for predicting PM status of 0.751 (95% CI, 0.703–0.799) in the training cohort and 0.802 (95% CI, 0.691–0.912) and 0.745 (95% CI, 0.683–0.806) in internal and external validation cohorts, respectively. Furthermore, when performing the stratified analysis according to clinicopathological risk factors, the Rad-score was still significantly associated with PM status in the training and internal and external validation cohorts (Tables S4–S6).

### Development of Radiomics Nomogram

**Figure 2:**
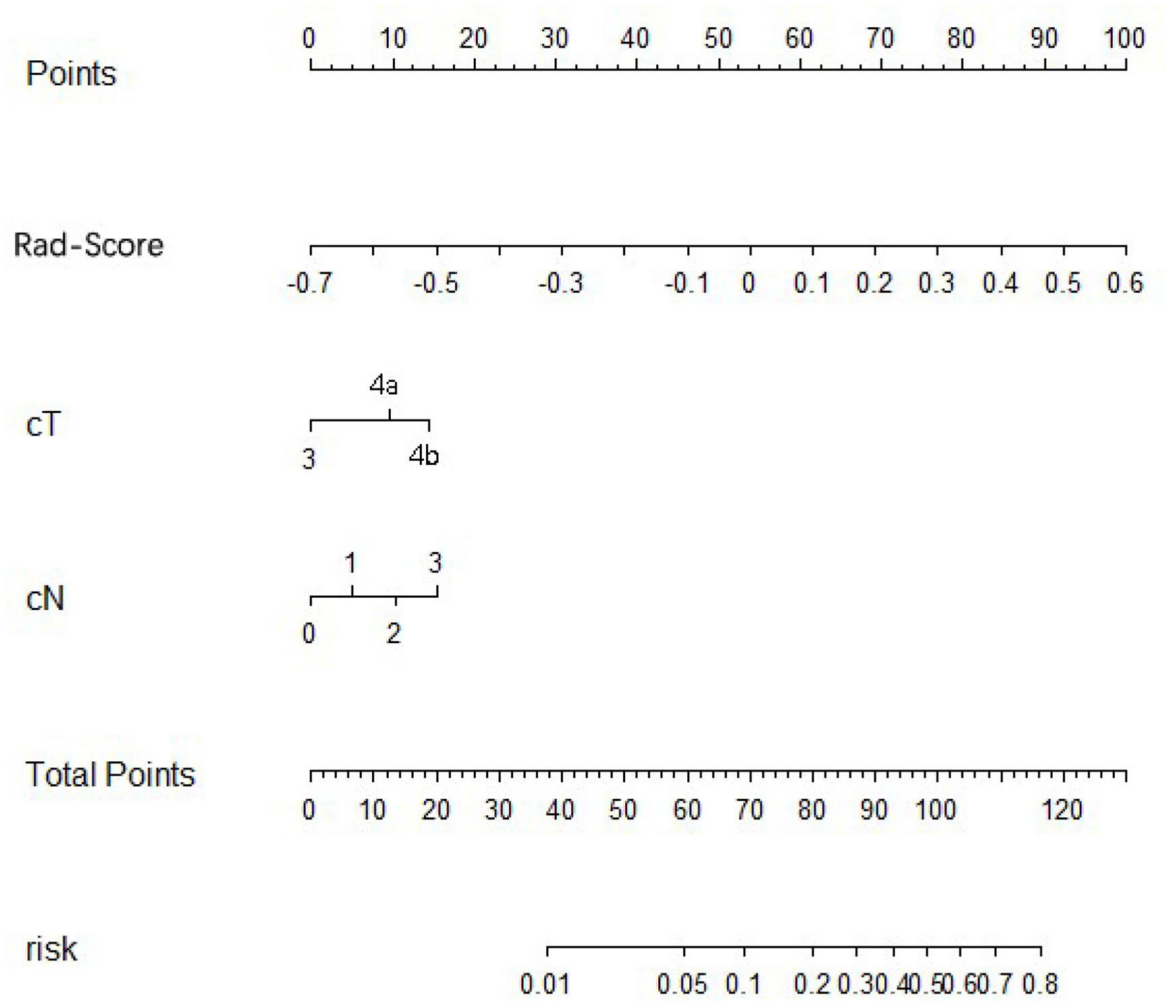
Development of radiomics nomogram in training cohort. The radiomics nomogram incorporating the radiomics signature and cT stage and cN stages was developed in the training cohort.

Using univariate analysis, we found that the radiomics signature was correlated with PM status significantly (Table S7). Variable values that demonstrated significance were used for multivariable analysis. In the training and internal and external validation cohorts, radiomics signature still remained an independent and powerful predictor for PM status in the multivariate logistic regression analysis (Table 2). According to the multivariate analysis, we developed a nomogram that integrated the radiomics signature and cT and cN stages in the training cohort (Figure 2). Using the nomogram, first draw a vertical line to the top point row to assign points for each variable; next, add the points from each variable together and drop a vertical line from the total points row to obtain the probability of PM status. For example, for a patient with a Rad-score of 0.4 and CT reported T4aN2 gastric cancer, the radiomics nomogram would predict a total score of more than 100, which indicates that the probability to suffer from peritoneal metastasis would be higher than 50%. The relationship between nomogram score and PM status is shown in Figure S7. Higher Nom-scores were found in PM(+) patients in the training cohorts and internal and external validation cohorts consistently (Figure S7).

**Table 2:** Multivariate relationship between Rad-score and preoperative clinicopathological characteristics with peritoneal metastasis in each cohort.

**Variables**	**PM(+) vs. PM(–)**					
	**OR (95%CI)**	***P***	**OR (95%CI)**	***P***	**OR (95%CI)**	***P***
	**Training cohort**		**Internal validation cohort**		**External validation cohort**	
**Rad-score**	**6.364 (3.387–11.959)**	**<0.0001**	**13.151 (2.175–79.512)**	**0.005**	**6.544 (2.924–14.642)**	**<0.0001**
**Location**						
Cardia	0.362 (0.156–0.838)	0.018	0.925 (0.052–16.590)	0.958	0.797 (0.271–2.339)	0.679
Body	0.803 (0.335–1.923)	0.623	2.581 (0.306–21.744)	0.383	0.667 (0.206–2.156)	0.499
Antrum	0.877 (0.394–1.951)	0.747	1.846 (0.224–15.206)	0.569	0.746 (0.295–1.890)	0.537
Whole	**Reference**		**Reference**		**Reference**	
**cT Stage**						
T3	0.300 (0.120–0.754)	0.01	0.150 (0.020–1.103)	0.062	/	0.997
T4a	0.684 (0.395–1.184)	0.175	0.183 (0.040–0.842)	0.029	0.221 (0.076–0.640)	0.005
T4b	**Reference**		**Reference**		**Reference**	
**cN Stage**						
N0	0.268 (0.126–0.571)	0.001	/	0.998	0.177 (0.047–0.665)	0.01
N1	0.617 (0.315–1.208)	0.159	0.085 (0.007–1.071)	0.057	0.466 (0.169–1.284)	0.14
N2	0.935 (0.499–1.753)	0.835	1.150 (0.287–4.612)	0.843	1.750 (0.799–3.833)	0.162
N3	**Reference**		**Reference**		**Reference**	

*PM(+), PM-positive status; PM(–), PM-negative status; CI, confidence interval; OR, odds ratio*.

### Validation of the Nomogram

**Figure 3:**
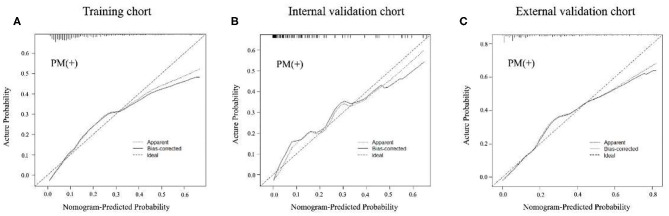
Calibration curves of radiomics nomogram of peritoneal metastasis (PM) status prediction in training and internal and external validation cohorts. The calibration curves described the calibration in agreement between predicted and observed outcome. The 45-degree reference line means a perfect calibration with the outcome by ideal model. The solid line is the performance of the nomogram, without correction for overfit. The dotted line is the bootstrap-corrected performance of the nomogram, with a scatter estimate for future accuracy. **(A–C)** Calibration curves in the training cohort, internal and external validation cohorts.

**Figure 4:**
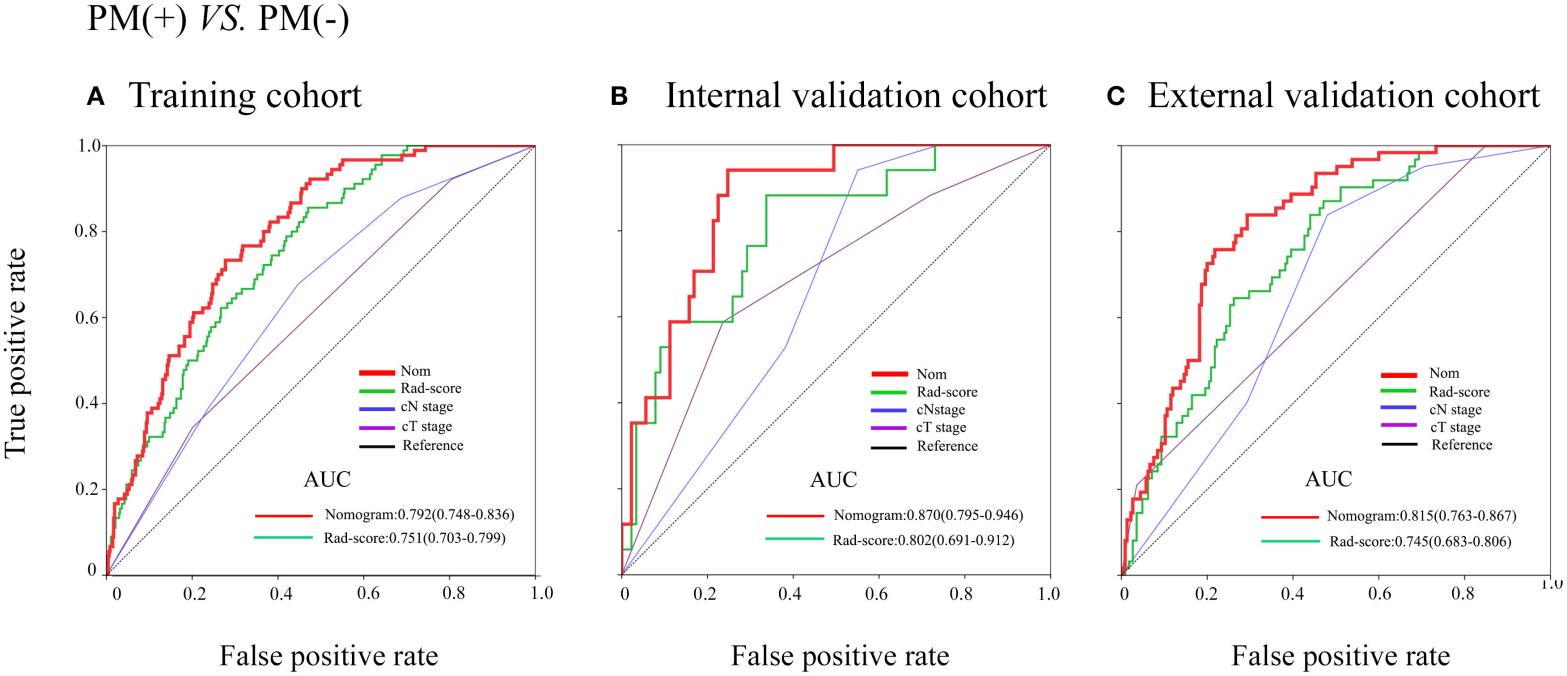
Receiver operating characteristic (ROC) curves of the models in training and internal and external validation cohorts. **(A–C)** ROC curves in the training cohort, internal and external cohorts.

**Figure 5:**
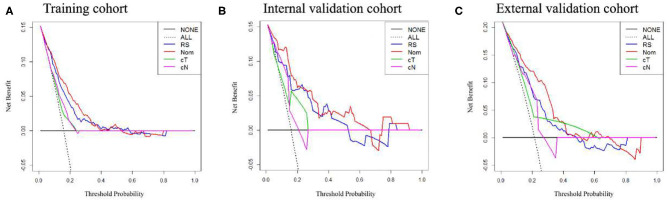
Decision curve analysis of the radiomics nomogram, Rad-score, and clinical *T* and *N* stage in each cohort. **(A–C)** Calibration curves in the training cohort, internal and external validation cohorts.

In the training cohort, an appropriate agreement between prediction and observation was yielded by the calibration curve of the radiomics nomogram (Figure 3A). ROC analysis showed good diagnostic performance of the Rad-score and nomogram in predicting gastric cancer peritoneal metastasis in each cohort (Table S8). The prediction performance of the model was moderate, with an AUC of 0.792 (95% CI, 0.748–0.836) in the training cohort (Figure 4A, Table 3, Table S8). In the validation cohort, it also displayed excellent prediction efficacy (Figures 3B,C), with AUCs of 0.870 (95% CI, 0.795–0.946) and 0.815 (95% CI, 0.763–0.867) in the internal and external validation cohorts, respectively (Figures 4B,C, Table 3, Table S8). The decision curve analysis for the nomogram in different cohorts are shown in Figure 5.

**Table 3:** Predictive accuracy of newly developed nomogram, Rad-score, and clinicopathological characteristics.

**Variables**	**AUC (95% CI)**
	**PM(+) vs. PM(–)**
**TRAINING COHORT**	
Nomogram	0.792 (0.748–0.836)
Rad-score	0.751 (0.703–0.799)
cN stage	0.639 (0.580–0.698)
cT stage	0.604 (0.542–0.667)
**INTERNAL VALIDATION COHORT**	
Nomogram	0.870 (0.795–0.946)
Rad-score	0.802 (0.691–0.912)
cN stage	0.669 (0.557–0.780)
cT stage	0.689 (0.548–0.830)
**DATION COHORT**	
Nomogram	0.815 (0.763–0.867)
Rad-score	0.745 (0.683–0.806)
cN stage	0.664 (0.597–0.731)
cT stage	0.647 (0.570–0.723)

*AUC, area under the receiver operating characteristic curve; CI, confidence interval; PM, peritoneal metastasis; Rad-score, radiomics score*.

## Discussion

In this study, we first established a radiomics signature based on four texture features. This PM-related radiomics signature was obviously correlated with PM and was an independent predictor of PM status in GC. Second, we constructed the preoperative individualized prediction of PM status by developing and validating a radiomics nomogram that incorporated the radiomics signature and cT and cN stages. Both of the nomogram and the radiomics signature can be used to assist clinicians to predict peritoneal metastasis non-invasively.

We excluded patients with pathological T1–T3 tumors because, compared to pathological T4, these parts of patients are less expected to have PM (6, 7). If we had included patients with pT1–pT3 tumors, the total incidence rate of PM would decrease, reducing the specificity and sensitivity of the model for predicting PM. Actually, those CT-reported T3 or T4 patients are more likely to have the risk of PM (9), and most of them were eventually conformed T3 or T4 stage but less even none T1 or T2. All of the cancer centers in this study were in the same situation.

The LASSO method is a powerful method for the regression of high-dimensional predictors (31, 32). In this study, we shrank the regression coefficients with the LASSO method to examine the predictor-outcome association, and as a result, 292 candidate radiomics features that were extracted from the primary CT image were reduced to 11 potential predictors. After addressing the collinearity, four PM-related features were selected for the construction of the radiomics signature.

Although CT is very popular and important in preoperative diagnosis, the accuracy of CT for preoperatively identifying PM status was very limited in patients with GC (9). PET-CT was a good method for predicting the LNM status preoperatively and had value on distant organ metastasis (33), and Findlay et al. also pointed out in their study that when staging patients with gastric cancer, 18F-fluorodeoxyglucose (18F-FDG) PET-CT could show useful information in identifying unsuspected metastasis (34); however, its accuracy for PM did not demonstrate an advantage over CT (33). Several studies have demonstrated that some clinicopathological factors, like CEA or CA19-9 level, size of the tumor, invasion depth, Borrmann type, and differentiation type, showed relationships with LNM (35). Some nomograms were developed for predicting LNM in patient with GC by using above clinicopathological factors, but outer validations still require to be applied on these nomograms. What is more, no particular nomogram has been used widely in clinical settings (17). Dong et al. found that PM was associated with the texture of the nearby peritoneum of tumor (36), but actually nearby peritoneum contains lots of positive-metastasis lymph nodes, which may increase the false positive rate of PM, as the PM is also strongly associated with lymph node stage (9, 17). This had also been proven in our study. Hence, CT texture of the nearby peritoneum of the tumor still needs further examination.

CT images contain many medical information, and nowadays, radiologists could easily acquire complementary anatomical information of human tissues and definite tumor visually from CT images. However, there remain large amount of digital information for precise analysis, and different analytical tools could dig out different kinds of information. Recently, the rise in deep learning in medical research arouse hot topic among researchers, especially in disease detection and diagnosis (37, 38). Our previous study found that deep-learning-based CT image signature could help in predicting survival for patients with GC and in identifying which patients could benefit from adjuvant chemotherapy (39). However, deep learning requires large amount of sample for training. Radiomics, which can convert digital medical images to mineable data and analyze these data to improve detection, diagnosis, stage, and prediction power, may help us to improve the accuracy of detecting PM status preoperatively (11, 12, 15). Giganti et al. reported that texture features from DW-MRI and CE-MDCT could be promising non-invasive biomarker in evaluating the prognosis of gastric cancer (40), suggesting that texture analysis from medical images could facilitate clinical decision. Recently, Liu et al. reported that venous CT radiomics analysis could provide interesting information for predicting occult PM in gastric cancer (41), and before this study, a radiomics signature that could predict LNM in colorectal cancer was developed (17); therefore, we aimed to present a predictive model for preoperative prediction of PM status by connecting the preoperative clinicopathological factors and the radiomics features.

In East Asian countries, radical gastrectomy with chemotherapy is the standard treatment for advanced GC, and the usefulness of neoadjuvant chemotherapy is recently being measured (42, 43). In some clinical trials, preoperative chemotherapy was performed in GC patients with extensive metastasis. Recently, study found that patients with peritoneal metastases of gastric cancer may benefit from cytoreductive surgery (CRS) and hyperthermic intraperitoneal chemotherapy (HIPEC) (44). Therefore, the radiomics nomogram for preoperatively predicting PM status may contribute to make an adequate preoperative medical decision and select patients who could benefit from above treatment.

Four-feature radiomics signature and two preoperative clinical factors (cT and cN stages, which are easily obtained from CT) are integrated in our radiomics nomogram. According to the nomogram, the status of the disease could be comprehensively reflected, and the accuracy of prediction could be obviously improved. Calibration plots and ROC analysis were used to validate the nomogram. The nomogram showed excellent prediction with a good calibration. What is more, high AUCs were demonstrated in our radiomics nomogram both in internal and external cohorts when predicting the PM status; this could provide more valuable information for determining the need for adjuvant therapy and the adequacy of surgical resection, thus aiding in pretreatment decision making.

In this study, our radiomics signature and nomogram could provide meaningful message for preoperatively predicting PM status. In the future, we will put a preoperative prediction model into effect to help provide proper surgical procedures or select candidates with high risk for laparoscopy exploration and treatment based on the comprehensive consideration of the information of the radiomics features.

However, there are still limitations in our study. Although the radiomics signature and nomogram could provide meaningful message for predicting PM, the nomograms were developed and externally validated in three retrospective data sets from two Chinese institutions; thus, these results need to be validated in a larger population with a multicenter and prospective study in the future, which could develop high-level evidence needed for clinical use. In addition, this predictive model is suitable for those who were preoperatively diagnosed cT3 or cT4, as these patients are more likely to have the risk of PM; however, clinical and pathological stages are sometimes inconsistent, especially in gastric cancer (45). For example, those who were diagnosed clinical T3 or T4 sometimes were found to be T1 or T2, and some patients who were diagnosed clinical T1 or T2 were confirmed T3 or T4 after surgery. For those who were pT3 or pT4 but were diagnosed cT1 or cT2, they would miss the opportunity to use this model for prediction. Furthermore, some serological matter like CA125 and HER-2 were not included in this study, as this study is retrospective and the above data were not available from each patient in that period.

In conclusion, this study demonstrated that the radiomics signature based on CT can be used as a predictor for predicting peritoneal metastasis in GC patients. Besides, this study revealed that the radiomics nomogram, which combined the clinicopathological risk factors with the radiomics signature, can be effectively used to promote the preoperative individualized prediction of PM status in patients with GC.

## Data Availability Statement

All datasets generated for this study are included in the article/Supplementary Material.

## Ethics Statement

The studies involving human participants were reviewed and approved by the Ethics Committee of Nanfang Hospital, Nanfang Hospital, Southern Medical University; the Ethics committee of Sun Yat-sen University Cancer Center, Sun Yat-sen University Cancer Center. Written informed consent for participation was not required for this study in accordance with the national legislation and the institutional requirements.

## Author Contributions

GL, YX, and ZZ: guarantor of the article. GL, YX, ZZ, YJ, WH, and KZ: conception and design. YJ, WH, KZ, CC, QY, ZH, SY, YH, JX, JY, and HL: collection and assembly of data. WH, YJ, RX, ZH, JX, ZS, and JY: data analysis and interpretation. All authors: manuscript writing and final approval of manuscript. All authors contributed to the article and approved the submitted version.

## Conflict of Interest

The authors declare that the research was conducted in the absence of any commercial or financial relationships that could be construed as a potential conflict of interest.
