# Supplementary material for: Radiomics Nomogram for Prediction of Peritoneal Metastasis in Patients With Gastric Cancer
Source: Front Oncol. 2020 Aug 20;10:1416. doi: 10.3389/fonc.2020.01416 (PMC7468436; doi:10.3389/fonc.2020.01416)
Supplement: Supplementary file 1 [file Data_Sheet_1.docx]

**­­­****Supplementary Materials**

1. **Supplementary Methods**
2. **Supplementary Reference**
3. **Supplementary Figures**
4. **Supplementary Tables**

**Supplementary Methods**

1. **Image Acquisition details**

The acquisition parameters are as follows: 120 kV; 150-190 mAs; 0.5- or 0.4-second rotation time; detector collimation: 8×2.5 mm or 64×0.625 mm; field of view, 350×350 mm; matrix, 512×512. After routine non-enhanced CT, arterial and portal venous-phase contrast-enhanced CT were performed after delays of 28 s and 60 s following intravenous administration of 90 - 100 ml of iodinated contrast material (Ultravist 370, Bayer Schering Pharma, Berlin, Germany) at a rate of 3.0 or 3.5 ml/s with a pump injector (Ulrich CT Plus 150, Ulrich Medical, Ulm, Germany). Contrast-enhanced CT was reconstructed with a reconstruction thickness of 2.5 mm. Portal venous phase CT images (thickness: 2.5 mm) were retrieved from the picture archiving and communication system (PACS) (Carestream, Canada) for image feature extraction because of well differentiation of the tumor tissue from the adjacent tissue.

2. **Inter-observer and intra-observer agreements of CT image feature extraction**

The inter-observer and intra-observer agreements of CT image feature extraction were initially analyzed with 100 randomly chosen images for ROI-based texture feature extraction by two experienced radiologists that both had clinical experience in abdominal CT study interpretation for more than 10 years. To evaluate whether the intra-observer agreement was appropriate, two radiologists repeated the extraction of texture features twice in a 4-week period with the same procedure. Then an independent samples t-test or Kruskal-Wallis H test was used appropriately to analyze the variability between the features extracted by the first radiologist (first time) and those by the second radiologist. And the same test was applied again between the twice-extracted features by the two radiologists respectively. Finally, we used the inter-class and intra-class correlation coefficients (ICCs) to access the intra-observer and inter-observer agreement of features extraction. An ICC greater than 0.75 presents good agreement.

***Results*** Convincing inter-observer and intra-observer agreements of the texture feature extraction were calculated. There was no statistically significant difference between the features of the two radiologists or between the twice-extracted features by the two radiologists, with *P* values ranging from 0.72 to 0.89. Based on the radiologists’ two measurements, the inter-observer ICCs were good, ranging from 0.75 to 0.94. The intra-observer ICCs of the two radiologists calculated based on the twice-extracted features ranged from 0.79 to 0.95 and 0.76 to 0.94. Therefore, all outcomes were based on the measurement of the first radiologist.

**3. Definition of image features**

- ***Intensity features-14***

where p(i) is the probability of occurrence of voxels with intensity i

1. CT_min: minimum of CT image
2. CT_max: maximum of CT image
3. CT_mean: mean of CT image
4. CT_median: median of CT image
5. CT_std: Standard Deviation of CT image
6. Hist_Skewness:

the asymmetry of the grey-level distribution in the histogram

1. Hist_Kurtosis:

reflects the shape of the grey-level distribution (peaked or flat) relative to a normal distribution.

1. Hist_Entropy:

$$entropy\_hist=-\sum_{i=1} p(i)\log_{2}[p(i)]$$

1. Hist_Consistency:

$$consistency\_ hist=-\sum_{i=1} {p\left( i \right)}^{2}$$

2. Hist_Energy:

$$energy\_hist=\sum_{i=1} p(i)^{2}$$

1. CT_range: the range of CT image
2. Hist_Var:

Variance of CT image

1. CT_RMS: root mean square, the quadratic mean, or the square root of the mean of squares of CT image
2. CT_MAD: Mean absolute deviation, the mean of the absolute deviations of CT image around the mean CT value.

- ***Shape features-9***

Shape features, describing the shape and size of the volume of interest. Let $M$ as the number of voxels in the tumor.

Using “regionprops” in Matlab.

1. Area: Actual number of pixels in the region, returned as a scalar.
2. Orientation: Angle between the x-axis and the major axis of the ellipse that has the same second-moments as the region, returned as a scalar. The value is in degrees, ranging from -90 degrees to 90 degrees.
3. Eccentricity: Eccentricity of the ellipse that has the same second-moments as the region, returned as a scalar.
4. Equivdiameter: Diameter of a circle with the same area as the region, returned as a scalar.sqrt(4*Area/pi)
5. Solidity: Proportion of the pixels in the convex hull that are also in the region, returned as a scalar.

Area/ConvexArea

1. Extent: Ratio of pixels in the region to pixels in the total bounding box, returned as a scalar. Area divided by the area of the bounding box
2. Eulernumber: Number of objects in the region minus the number of holes in those objects.
3. Perimeter: Distance around the boundary of the region.

- ***Gray Level Co-occurrence Matrix-based features (GLCM)-26***

Gray level co-occurrence matrix-based features, as described by study. The element $P(i,j)$ of normalized co-occurrence matrix represent the number of times that intensity $\text{i}$ and $\text{j}$ appeared in two voxels separated by distance D in direction $\theta$. The co-occurrence matrix is given by:

$$P(i,j)=\#\left\{(I(\text{x},\text{y},\text{z})=\text{i},\text{I}(\text{k},\text{l},\text{m})=\text{j})\text{ | D},\theta\right\}$$

where # represents the number of times, $I$ represents the voxel intensity, $(\text{x},\text{y},\text{z})$ and $(\text{k},\text{l},\text{m})$ are the coordinates (positions) of two different voxels, the direction vector is thus determined by $(\text{k},\text{l},\text{m})-(\text{x},\text{y},\text{z})$, $N_{g}$ is the number of discrete intensity levels in the image, and $\mu$ is the mean of $P(i,j)$. The feature is derived by considering all the 13 directions simultaneously, thus arriving at a single matrix.

Let us define:

$$\mu_{x}\text{=}\sum_{\text{i}=1}^{\text{N}_{\text{g}}} i\sum_{j=1}^{Ng} P(i,j)$$

$$\mu_{\text{y}}\text{=}\sum_{\text{j}=1}^{\text{N}_{\text{g}}} j\sum_{i=1}^{Ng} P(i,j)$$

$$\sigma_{x}\text{=}\sqrt{\sum_{\text{i}=1}^{\text{N}_{\text{g}}} (i-\mu_{x})^{2}\sum_{j=1}^{Ng} P(i,j)}$$

$$\sigma_{y}\text{=}\sqrt{\sum_{j=1}^{\text{N}_{\text{g}}} (j-\mu_{\text{y}})^{2}\sum_{i=1}^{Ng} P(i,j)}$$

$$p_{x}\left(i\right)=\sum_{j=1}^{N_{g}} P\left(i,j\right)$$

$$p_{y}\left(j\right)=\sum_{i=1}^{N_{g}} P\left(i,j\right)$$

$$p_{x+y}\left(k\right)=\sum_{i=1}^{N_{g}} \sum_{j=1}^{N_{g}} P\left(i,j\right), i+j=k,k=2,3,\ldots2N_{g}$$

$$p_{x-y}\left(k\right)=\sum_{i=1}^{N_{g}} \sum_{j=1}^{N_{g}} P\left(i,j\right), \left|i-j\right|=k,k=0,1,\ldots N_{g}-1$$

$$\mu_{x\text{-y}}\text{=}\sum_{k=0}^{\text{N}_{\text{g}}-1} kP_{\text{x-y}}(k)$$

$$HXY1=-\sum_{i=1}^{N_{g}} \sum_{j=1}^{N_{g}} P\left(i,j\right)\log_{2}\left(p_{x}\left(i\right)p_{y}\left(j\right)\right)$$

$$HXY2=-\sum_{i=1}^{N_{g}} \sum_{j=1}^{N_{g}} P_{x}\left(i\right)P_{y}\left(j\right)\log_{2}\left(p_{x}\left(i\right)p_{y}\left(j\right)\right)$$

$$HX=-\sum_{i=1}^{N_{g}} p_{x}(i)\log_{2}[p_{x}(i)]$$

$$HY=-\sum_{j=1}^{N_{g}} p_{y}(j)\log_{2}[p_{y}(j)]$$

$$H=-\sum_{i=1}^{N_{g}} \sum_{j=1}^{N_{g}} p(i,j)\log_{2}[p(i,j)]$$

The various radiomics features based on the co-occurrence matrix are then defined as:

1. Auto correlation (AutoCorrelation):

$$auto correlation=\sum_{i=1}^{N_{g}} \sum_{j=1}^{N_{g}} ijp\left(i,j\right)$$

1. Cluster prominence (ClusterPro):

$$cluster prominence=\sum_{i=1}^{N_{g}} \sum_{j=1}^{N_{g}} \left[i+j-\mu_{x}-\mu_{y}\right]^{4}P\left(i,j\right)$$

1. Cluster shade (ClusterShade):

$$cluster shade=\sum_{i=1}^{N_{g}} \sum_{j=1}^{N_{g}} \left[i+j-\mu_{x}-\mu_{y}\right]^{3}P\left(i,j\right)$$

1. Cluster tendency (ClusterTen):

$$cluster tendency=\sum_{i=1}^{N_{g}} \sum_{j=1}^{N_{g}} \left[i+j-\mu_{x}-\mu_{y}\right]^{2}P\left(i,j\right)$$

1. Contrast:

$$contrast=\sum_{i=1}^{N_{g}} \sum_{j=1}^{N_{g}} \left|i-j\right|^{2}P\left(i,j\right)$$

1. Correlation:

$$correlation=\sum_{i=1}^{N_{g}} \sum_{j=1}^{N_{g}} \frac{(i-\mu_{x})(j-\mu_{y})p(i,j)}{\sigma_{x}\sigma_{y}}\text{=}\frac{\sum_{i=1}^{N_{g}} \sum_{j=1}^{N_{g}} ijP\left(i,j\right)-\mu_{x}\mu_{y}}{\sigma_{x}\sigma_{y}}$$

1. Difference entropy (DiffEntropy):

$$difference entropy=-\sum_{k=0}^{N_{g}-1} P_{x-y}\left(k\right)\log_{2}\left[P_{x-y}\left(k\right)\right]$$

1. Difference Variance (DiffVar):

$$difference variance=-\sum_{k=0}^{N_{g}-1} \left(k-\mu_{x-y}\right)^{2}P_{x-y}\left(k\right)$$

$$\mu_{x\text{-y}}\text{=}\sum_{k=0}^{\text{N}_{\text{g}}-1} kP_{\text{x-y}}(k)$$

1. Dissimilarity:

$$dissimilarity=\sum_{i=1}^{N_{g}} \sum_{j=1}^{N_{g}} \left|i-j\right|P\left(i,j\right)$$

1. Energy, called Uniformity in(1), also called Angular second moment in(2):

$$energy=\sum_{i=1}^{N_{g}} \sum_{j=1}^{N_{g}} \left[P\left(i,j\right)\right]^{2}$$

1. Entropy:

$$entropy=-\sum_{i=1}^{N_{g}} \sum_{j=1}^{N_{g}} P\left(i,j\right)\log_{2}\left[P\left(i,j\right)\right]$$

1. Homogeneity, also called Inverse difference in(1) :

$$homogeneity =\sum_{i=1}^{N_{g}} \sum_{j=1}^{N_{g}} \frac{P\left(i,j\right)}{1+\left|i-j\right|}$$

1. Informational measure of correlation 1 (IMC1):

$$IMC1=\frac{H-HXY1}{\max\left\{HX,HY\right\}}$$

Where *HX and HY* are the entropies of $p_{x}(i)$ and $p_{y}(\text{j})$.

1. Informational measure of correlation 2 (IMC2):

$$IMC2=\sqrt{1-e^{-2\left(HXY2-H\right)}}$$

where *H* is the entropy of $p(i,j)$.

1. Inverse Difference Normalized (IDN):

$$IDN=\sum_{i=1}^{N_{g}} \sum_{j=1}^{N_{g}} \frac{P\left(i,j\right)}{1+\left(\frac{\left|i-j\right|}{N}\right)}$$

1. Inverse Difference Moment Normalized (IDMN):

$$IDMN=\sum_{i=1}^{N_{g}} \sum_{j=1}^{N_{g}} \frac{P\left(i,j\right)}{1+\left(\frac{\left|i-j\right|^{2}}{N^{2}}\right)}$$

1. Inverse variance (InVar):

$$inverse variance=\sum_{i=1}^{N_{g}} \sum_{j=1}^{N_{g}} \frac{P\left(i,j\right)}{\left|i-j\right|^{2}},i\neq j$$

1. Maximum probability (MaxPossilility):

$$maximum probability=\max\left\{P\left(i,j\right)\right\}$$

1. Sum average2:

$$sum average2=\sum_{k=2}^{2N_{g}} \left[kP_{x+y}\left(k\right)\right]$$

1. Sum entropy (SumEntropy):

$$sum entropy=-\sum_{k=2}^{2N_{g}} P_{x+y}\left(k\right)\log_{2}\left[P_{x+y}\left(k\right)\right]$$

1. Sum variance (SumVar):

$$sum variance=\sum_{k=2}^{2N_{g}} \left(k-SA\right)^{2}P_{x+y}\left(k\right)$$

where SA is Sum average2.

1. Variance:

$$variance=\frac{1}{N_{g}\times N_{g}}\sum_{i=1}^{N_{g}} \sum_{j=1}^{N_{g}} [(i-\mu_{x})^{2}+(j-\mu_{y})^{2}]p(i,j)$$

- ***Gray Level Run Length Matrix-based features (GLRLM)-13***

Gray level run length matrix-based features are described by Galloway et al.(3). The element of GLRLM $P(i,j)$ counts the number of runs $j$ with collinearly adjacent pixels having the same gray level intensity $i$ as follows:

$$P(i,j)=\left\{\text{j | }I_{1}=\text{i},I_{2}=\text{i},...,I_{j}=\text{i}\right\}$$

where $I_{1},I_{2},...,I_{j}$ are collinearly adjacent voxels.

The GLRLM feature value was derived by considering all the 13 directions simultaneously, thus arriving at a single matrix. Let $P(i,j)$ be the $(i,j)\text{ }th$ entry in the given run-length matrix, $N_{g}$ the number of discrete intensity values in the image, $N_{r}$ the number of different run lengths, $N_{p}$ the number of voxels in the image, and the entry $(i,j)$ of the normalized GLRLM defined as:

$$p(i,j)=\frac{P(i,j)}{\sum_{i=1}^{N_{g}} \sum_{j=1}^{N_{r}} P(i,j)}$$

$$\mu_{i}=\sum_{i=1}^{N_{g}} i\sum_{j=1}^{N_{r}} p(i,j)$$

$$\mu_{j}=\sum_{j=1}^{N_{r}} j\sum_{i=1}^{N_{g}} p(i,j)$$

Then the GLRLM-based features are defined as:

1. Short Run Emphasis (SRE):

$$SRE=\sum_{i=1}^{N_{g}} \sum_{j=1}^{N_{r}} \left[\frac{p\left(i,j\right)}{j^{2}}\right]$$

1. Long Run Emphasis (LRE):

$$LRE=\sum_{i=1}^{N_{g}} \sum_{j=1}^{N_{r}} j^{2}p\left(i,j\right)$$

1. Gray Level Non-Uniformity (GLN):

$$GLN=\sum_{i=1}^{N_{g}} \left[\sum_{j=1}^{N_{r}} p\left(i,j\right)\right]^{2}$$

1. Run Length Non-Uniformity (RLN):

$$RLN=\sum_{j=1}^{N_{r}} \left[\sum_{i=1}^{N_{g}} p\left(i,j\right)\right]^{2}$$

1. Run Percentage (RP):

$$RP=\sum_{i=1}^{N_{g}} \sum_{j=1}^{N_{r}} \frac{p\left(i,j\right)}{N_{p}}$$

1. Low Gray Level Run Emphasis (LGRE):

$$LGRE=\sum_{i=1}^{N_{g}} \sum_{j=1}^{N_{r}} \left[\frac{p\left(i,j\right)}{i^{2}}\right]$$

1. High Gray Level Run Emphasis (HGRE):

$$HGRE=\sum_{i=1}^{N_{g}} \sum_{j=1}^{N_{r}} i^{2}p\left(i,j\right)$$

1. Short Run Low Gray Level Emphasis (SRLGE):

$$SRLGE=\sum_{i=1}^{N_{g}} \sum_{j=1}^{N_{r}} \left[\frac{p\left(i,j\right)}{i^{2}j^{2}}\right]$$

1. Short Run High Gray Level Emphasis (SRHGE):

$$SRHGE=\sum_{i=1}^{N_{g}} \sum_{j=1}^{N_{r}} \left[\frac{p\left(i,j\right)i^{2}}{j^{2}}\right]$$

1. Long Run Low Gray Level Emphasis (LRLGE):

$$LRLGE=\sum_{i=1}^{N_{g}} \sum_{j=1}^{N_{r}} \left[\frac{p\left(i,j\right)j^{2}}{i^{2}}\right]$$

1. Long Run High Gray Level Emphasis (LRHGE):

$$LRHGE=\sum_{i=1}^{N_{g}} \sum_{j=1}^{N_{r}} p\left(i,j\right)i^{2}j^{2}$$

1. Gray Level Variance (GLV)

$$GLV=\frac{1}{N_{g}\times N_{r}}\sum_{i=1}^{N_{g}} \sum_{j=1}^{N_{r}} (ip(i,j)-\mu_{i})^{2}$$

1. Run length Variance (RLV)

$$RLV=\frac{1}{N_{g}\times N_{r}}\sum_{i=1}^{N_{g}} \sum_{j=1}^{N_{r}} (jp(i,j)-\mu_{j})^{2}$$

- ***Gray Level Size Zone Matrix-based features (GLSZM)-13***

Gray-level size-zone matrix-based features, was described in [1]. GLSZM describes the number of a certain size zone $j$ having same intensity $i$ within N-connected neighbors in a 3D space as follows:

$$P(i,j)=\left\{\text{j | }I_{1}=\text{i},I_{2}=\text{i},...,I_{j}=\text{i}\right\}$$

where voxels $I_{1},I_{2},...,I_{j}$ are within N-connected neighbors (N=26).

Let $P(i,j)$ be the $(i,j)th$ entry in the given size-zone matrix, $N_{g}$ the number of discrete intensity values in the image, $N_{z}$ the size of the largest homogeneous region in the volume of interest, and $N_{\alpha}$ the number homogeneous zones in the image. The entry $(i,j)$ of the GLSZM are then normalized as:

$$p(i,j)=\frac{P(i,j)}{\sum_{i=1}^{N_{g}} \sum_{j=1}^{N_{z}} P(i,j)}$$

$$\mu_{i}=\sum_{i=1}^{N_{g}} i\sum_{j=1}^{N_{z}} p(i,j)$$

$$\mu_{j}=\sum_{j=1}^{N_{z}} j\sum_{i=1}^{N_{g}} p(i,j)$$

The GLSZM-based features are then defined as:

1. Small Zone Emphasis (SZE):

$$SZE=\sum_{i=1}^{N_{g}} \sum_{j=1}^{N_{z}} \left[\frac{p\left(i,j\right)}{j^{2}}\right]$$

1. Large Zone Emphasis (LZE):

$$LZE=\sum_{i=1}^{N_{g}} \sum_{j=1}^{N_{z}} j^{2}p\left(i,j\right)$$

1. Gray Level Non-uniformity (GLN) also called Intensity Variability (IV) in(4):

$$GLN=\sum_{i=1}^{N_{g}} \left[\sum_{j=1}^{N_{z}} p\left(i,j\right)\right]^{2}$$

1. Zone Size Non-uniformity (ZSN) also called Size Zone Variability (SZV) in(4):

$$ZSN=\sum_{j=1}^{N_{z}} \left[\sum_{i=1}^{N_{g}} p\left(i,j\right)\right]^{2}$$

1. Zone Percentage (ZP):

$$ZP=\sum_{i=1}^{N_{g}} \sum_{j=1}^{N_{z}} \frac{p\left(i,j\right)}{N_{\alpha}}$$

1. Low Gray Level Zone Emphasis (LGZE) also called Low Intensity Emphasis (LIE) in^4^:

$$LGZE=\sum_{i=1}^{N_{g}} \sum_{j=1}^{N_{z}} \left[\frac{p\left(i,j\right)}{i^{2}}\right]$$

1. High Gray level Zone Emphasis (HGZE) also called High Intensity Emphasis (HIE) in(4):

$$HGZE=\sum_{i=1}^{N_{g}} \sum_{j=1}^{N_{z}} i^{2}p\left(i,j\right)$$

1. Small Zone Low Gray Level Emphasis (SZLGE) also called Low Intensity Small Area Emphasis (LISAE) in(4):

$$SZLGE=\sum_{i=1}^{N_{g}} \sum_{j=1}^{N_{z}} \left[\frac{p\left(i,j\right)}{i^{2}j^{2}}\right]$$

1. Small Zone High Gray-Level Emphasis (SZHGE) also called High Intensity Small Area Emphasis (HISAE) in(4):

$$SZHGE=\sum_{i=1}^{N_{g}} \sum_{j=1}^{N_{z}} \left[\frac{p\left(i,j\right)i^{2}}{j^{2}}\right]$$

1. Large Zone Low Gray-Level Emphasis (LZLGE) also called Low Intensity Large Area Emphasis (LILAE) in^4^:

$$LZLGE=\sum_{i=1}^{N_{g}} \sum_{j=1}^{N_{z}} \left[\frac{p\left(i,j\right)j^{2}}{i^{2}}\right]$$

1. Large Zone High Gray-Level Emphasis (LZHGE) also called High Intensity Large Area Emphasis (HILAE) in(4):

$$LZHGE=\sum_{i=1}^{N_{g}} \sum_{j=1}^{N_{z}} p\left(i,j\right)i^{2}j^{2}$$

1. Gray Level Variance (GLV)

$$GLV=\frac{1}{N_{g}\times N_{z}}\sum_{i=1}^{N_{g}} \sum_{j=1}^{N_{z}} (ip(i,j)-\mu_{i})^{2}$$

1. Zone Size Variance (ZSV)

$$ZSV=\frac{1}{N_{g}\times N_{z}}\sum_{i=1}^{N_{g}} \sum_{j=1}^{N_{z}} (jp(i,j)-\mu_{j})^{2}$$

where zone aforesaid also called area in(4).

- ***Neighborhood Gray Tone Difference Matrix–based features (NGTDM)-5***

NGTDM is a column matrix(5). Denote the$i^{th}$entry of the NGTDM as$P(i)$, defined as:

$$P(i)=\left\{\begin{matrix} \sum_{i\in\left\{\left.N_{i}\right\}\right.} \left|i-\overline{A_{i}}\right|\text{ }if\text{ }N_{i}\text{>0,} \\ 0\text{ }otherwise. \end{matrix}\right.$$

where $\{N_{i}\}$ is the set of all voxels with gray-level $i$ in tumor volume (including the peripheral region), $N_{i}$ is the number of voxels with gray-level $i$ in tumor volume, and $A_{i}$ is the average gray level of the $M$ connected neighbors around a center voxel $V(i,j,k)$ with gray level $i$. Also, we have

$$\overline{A_{i}}=\overline{A(}j,k,l)=\frac{1}{M}\sum_{m=-d}^{d} \sum_{n=-d}^{d} \sum_{s=-d}^{d} V(j+m,k+n,l+s),(m,n,l)\neq(0,0,0)$$

where $d=1$, specifies the window size as $3\times3\times3$, and $M=(2d+1)^{3}-1$. The quantity $n_{i}=\frac{N_{i}}{N}$ is also defined, where $N$ is the total number of voxels in tumor volume. The NGTDM-based features are then defined as:

1. Coarseness:

$$coarseness=[\varepsilon+\sum_{i=1}^{N_{g}} n_{i}P(i)]^{-1}$$

where $\varepsilon$ is a small number to prevent coarseness becoming infinite, *N_g_* the number of discrete intensity values in the image.

1. Contrast:

$$contrast=[\frac{1}{N_{g}\times(N_{g}-1)}\sum_{i=1}^{N_{g}} \sum_{j=1}^{N_{g}} n_{i}n_{j}(i-j)^{2}]\times[\frac{1}{N}\sum_{i=1}^{N_{g}} P(i)]$$

1. Busyness:

$$busyness=\frac{\sum_{i=1}^{N_{g}} n_{i}P(i)}{\sum_{i=1}^{N_{g}} \sum_{j=1}^{N_{g}} \left|in_{i}-jn_{j}\right|},n_{i}\neq0,n_{j}\neq0$$

1. Complexity:

$$complexity=\sum_{i=1}^{N_{g}} \sum_{j=1}^{N_{g}} \frac{\left|i-j\right|\left[n_{i}P(i)+n_{j}P(j)\right]}{N(n_{i}+n_{j})},n_{i}\neq0,n_{j}\neq0$$

1. Strength:

$$strength=\frac{\sum_{i=1}^{N_{g}} \sum_{j=1}^{N_{g}} (n_{i}\text{+}n_{j})(i-j)^{2}}{\varepsilon+\sum_{i=1}^{N_{g}} P(i)},n_{i}\neq0,n_{j}\neq0$$

where $\varepsilon$ is a small number to prevent strength becoming infinite.

**4. Construction of Radiomics Signature using LASSO Logistic Regression Model**

The least absolute shrinkage and selection operator method (LASSO) is a popular method for regression of high-dimensional predictors(6, 7). The method uses an L1 penalty to shrink some regression coefficients to exactly zero. We selected λ via 1-SE (standard error) criteria, i.e., the optimal λ is the largest value for which the partial likelihood deviance is within one SE of the smallest value of partial likelihood deviance. Thus, we plotted the partial likelihood deviance versus log (λ), where λ is the tuning parameter. A value λ = 0.03907685 with log (λ) = -3.242225 was chosen by cross-validation via the 1-SE criteria. A vertical line was drawn at log (λ) = -3.242225, which corresponds to the optimal value λ = 0.03907685 (**Figure S2**). The optimal tuning parameter resulted in eleven non-zero coefficients. Eleven features (Eccentricity, Extent, GLCM_IMC1-0.5, GLCM_MaximumProbability-0.5, GLCM_IMC1-1, GLCM_MaximumProbability-1, GLCM_IMC1-1.5, GLCM_MaximumProbability-1.5, GLCM_IMC1-2, GLCM_MaximumProbability-2 and GLCM_IMC1-2.5, with coefficients -1.429359e-03, 1.232216e-02, -9.887834e-02, 8.977322e-02, -4.812247e-05, 1.858582e-05, -5.324134e-06, 1.433833e-06, -5.890471e-07, 7.418039e-08 and -6.517051e-08, respectively) were selected in the LASSO logistic regression model (**Figure S2B**). In order to detect and address the collinearity among variables, we used a scatterplot correlation matrix with Person correlation coefficient to investigate the interrelationship among clinical characteristics and these 11 features in the training cohort (**Figure S3**). After removing the features that have a correlation coefficient that higher than 0.80, four PM-related features were selected into Radiomics score (Rad-score) calculation formula for model development. The four features were Eccentricity, Extent, GLCM_IMC1-0.5 and GLCM_MaximumProbability-0.5, with coefficients of -1.429359e-03, 1.232216e-02, -9.887834e-02 and 8.977322e-02, respectively.

**5. R Software Packages Used for Statistical Analysis**

LASSO logistic regression was performed using the “glmnet” package. Nomogram and calibration plots were done with the “rms” package. Decision curve analysis was performed with the function of “dca.R”.

**6.** **Radiomics score (Rad-score)** **calculation formula:**

**Rad-score** = -1.429359e-03 * Eccentricity + 1.232216e-02 * Extent - 9.887834e-02 * GLCM_IMC1-0.5 + 8.977322e-02 * GLCM_MaximumProbability-0.5

**Supplementary Reference**

**References:**

1. Gomez W, Pereira WC and Infantosi AF Analysis of co-occurrence texture statistics as a function of gray-level quantization for classifying breast ultrasound. *IEEE Trans Med Imaging* (2012) 31:1889-99. doi:10.1109/TMI.2012.2206398

2. Lee J, Jain R, Khalil K, Griffith B, Bosca R and Rao G, et al. Texture Feature Ratios from Relative CBV Maps of Perfusion MRI Are Associated with Patient Survival in Glioblastoma. *AJNR Am J Neuroradiol* (2016) 37:37-43. doi:10.3174/ajnr.A4534

3. Galloway MM Texture analysis using grey level run lengths. *NASA STI/Recon Technical Report N* (1974) 75.

4. Leijenaar RT, Carvalho S, Velazquez ER, van Elmpt WJ, Parmar C and Hoekstra OS, et al. Stability of FDG-PET Radiomics features: an integrated analysis of test-retest and inter-observer variability. *Acta Oncol* (2013) 52:1391-7. doi:10.3109/0284186X.2013.812798

5. Amadasun M and King R Textural features corresponding to textural properties. *IEEE Transactions on systems, man, and Cybernetics* (1989) 19:1264-1274.

6. Jiang Y, Zhang Q, Hu Y, Li T, Yu J and Zhao L, et al. ImmunoScore Signature: A Prognostic and Predictive Tool in Gastric Cancer. *Ann Surg* (2018) 267:504-513. doi:10.1097/SLA.0000000000002116

7. Tibshirani R The lasso method for variable selection in the Cox model. *Stat Med* (1997) 16:385-95.


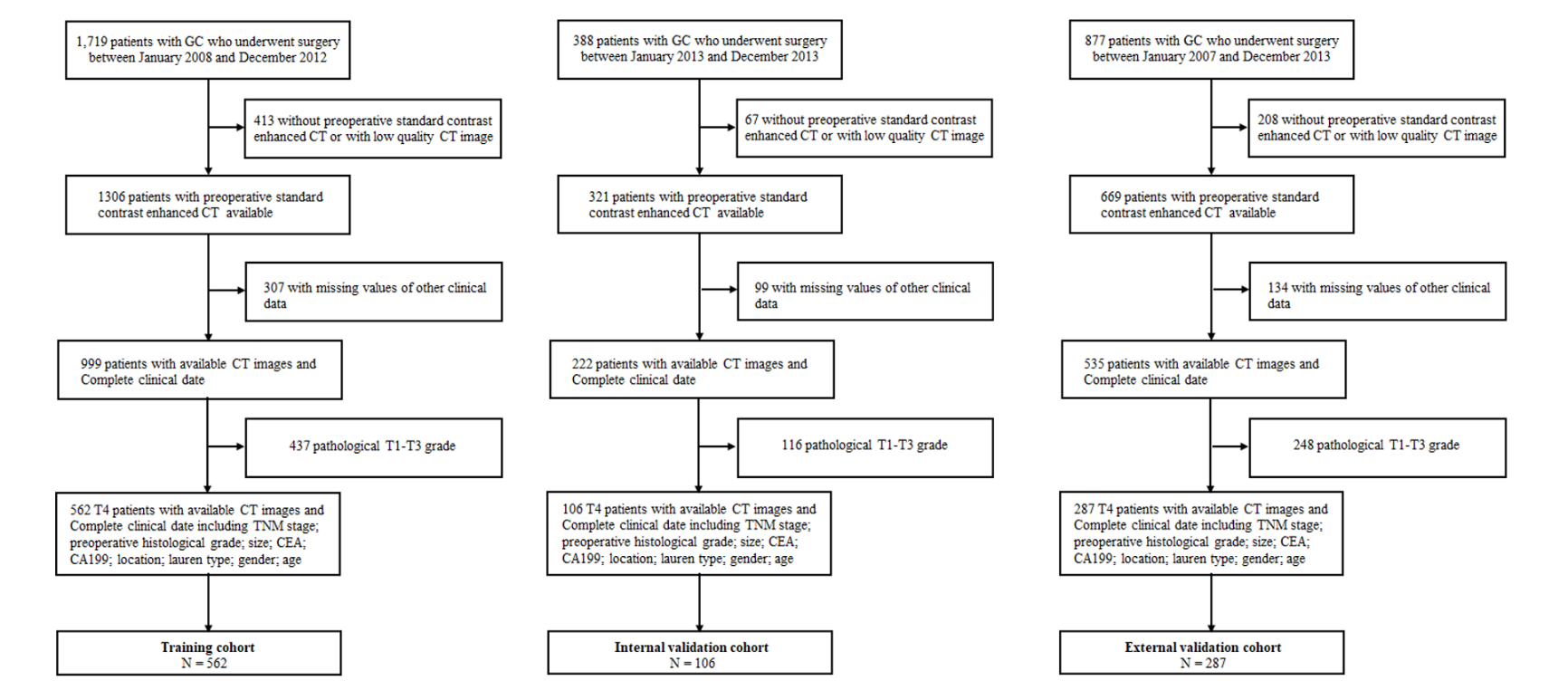


**Figure S1. Flow diagram of study population.**


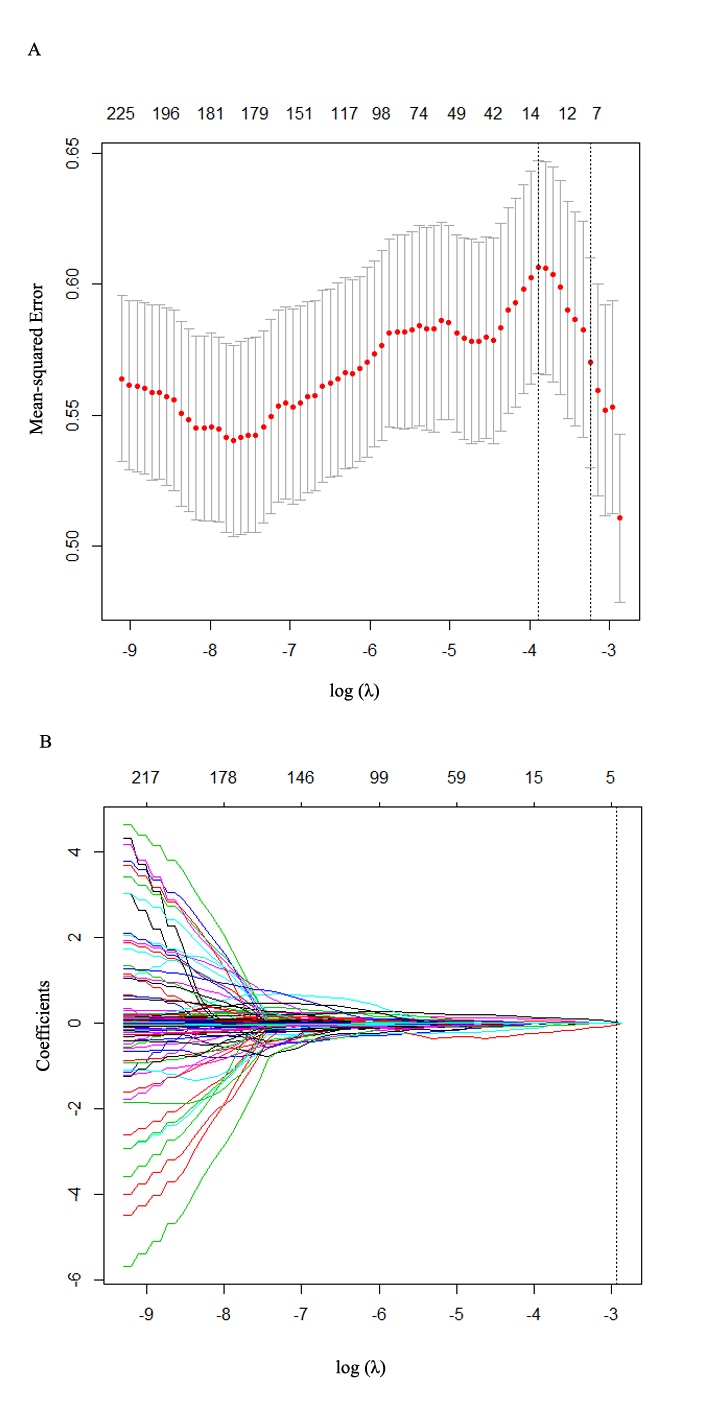


**Figure S2. Texture feature selection using the least absolute shrinkage and selection operator (LASSO) logistic regression model.** (A) Tuning parameter (λ) selection in the LASSO model used 10-fold cross-validation via minimum criteria. The partial likelihood deviance (PLD) curve was plotted versus log (λ). Dotted vertical lines were drawn at the optimal values by using the minimum criteria and 1 standard error of the minimum criteria (the 1-SE criteria). A λ value of 0.03907685, with log (λ) of -3.242225 was chosen (1-SE criteria) according to 10-fold cross-validation. (B) LASSO coefficient profiles of the 292 texture features. A coefficient profile plot was produced against the log (λ) sequence. A vertical line was drawn at the value selected using 10-fold cross-validation, where optimal λ resulted in eleven nonzero coefficients.


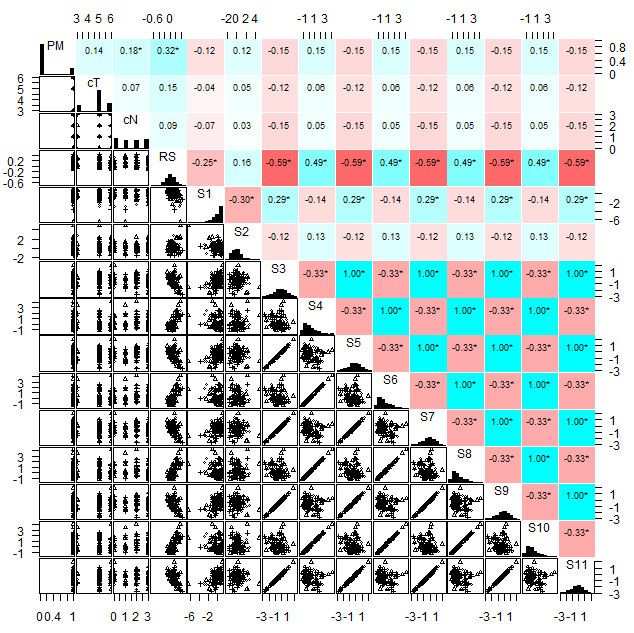


**Figure S3.** **Scatterplot matrix of the interrelationship among primary selected features and clinical characteristics in the training cohort.** PM, peritoneal metastasis status; RS, Rad-score; cT, CT-reported T stage; cN, CT-reported N stage; S1-S11, primary features selected by LASSO (Eccentricity, Extent, GLCM_IMC1-0.5, GLCM_MaximumProbability-0.5, GLCM_IMC1-1, GLCM_MaximumProbability-1, GLCM_IMC1-1.5, GLCM_MaximumProbability-1.5, GLCM_IMC1-2, GLCM_MaximumProbability-2 and GLCM_IMC1-2.5).


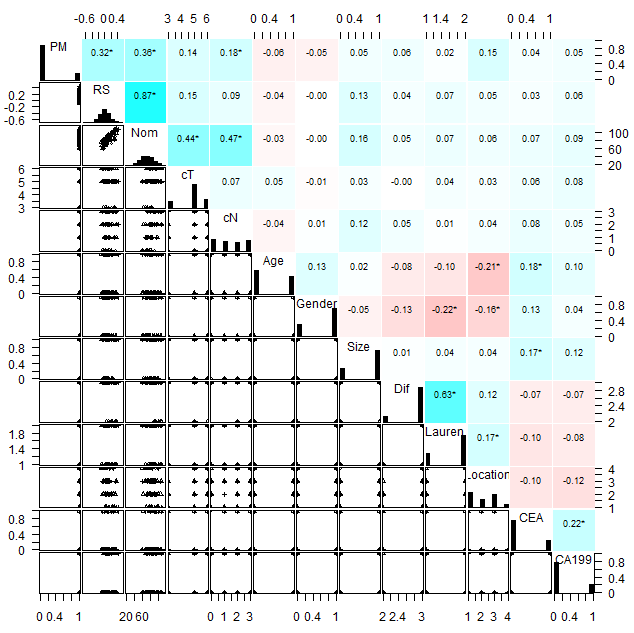


**Figure S4. S****catterplot matrix of the interrelationship among Rad-score, Nomogram and clinical characteristics in the training cohort.**  The values denote Pearson correlation coefficients, with numbers closer to 1 identifying a better correlation. *p<0.0001. PM, peritoneal metastasis status; RS, Rad-score; Nom, Nomogram; cT, CT-reported T stage; cN, CT-reported N stage; Dif, differentiation.


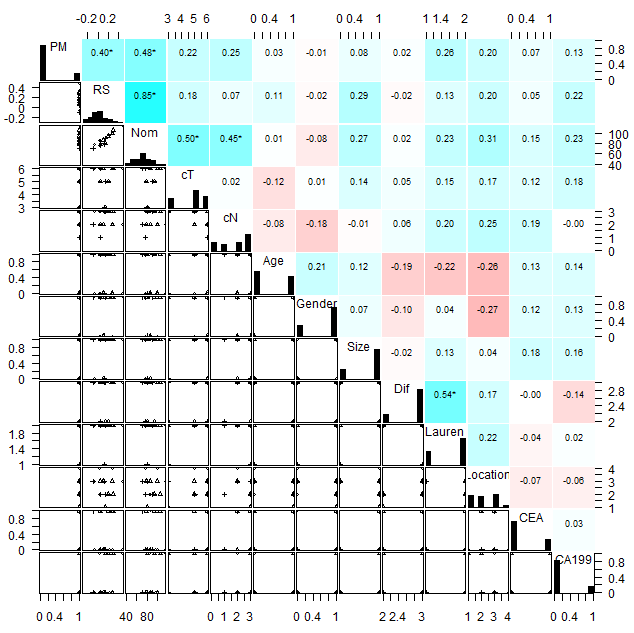


**Figure S5.** **Scatterplot matrix of the interrelationship among Rad-score, Nomogram and clinical characteristics in the internal validation cohort.** The values denote Pearson correlation coefficients, with numbers closer to 1 identifying a better correlation. *p<0.0001. PM, peritoneal metastasis status; RS, Rad-score; Nom, Nomogram; cT, CT-reported T stage; cN, CT-reported N stage; Dif, differentiation.


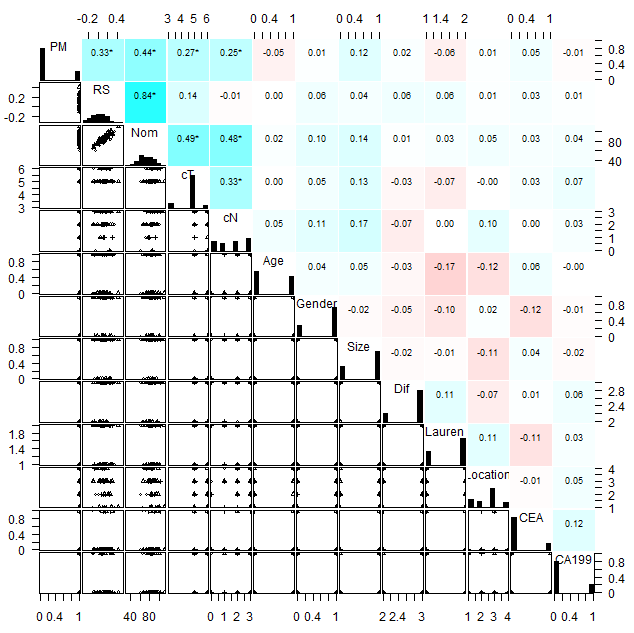


**Figure S6. Scatterplot matrix of the interrelationship among Rad-score, Nomogram and clinical characteristics in the external validation cohort.** The values denote Pearson correlation coefficients, with numbers closer to 1 identifying a better correlation. *p<0.0001. PM, peritoneal metastasis status; RS, Rad-score; Nom, Nomogram; cT, CT-reported T stage; cN, CT-reported N stage; Dif, differentiation.


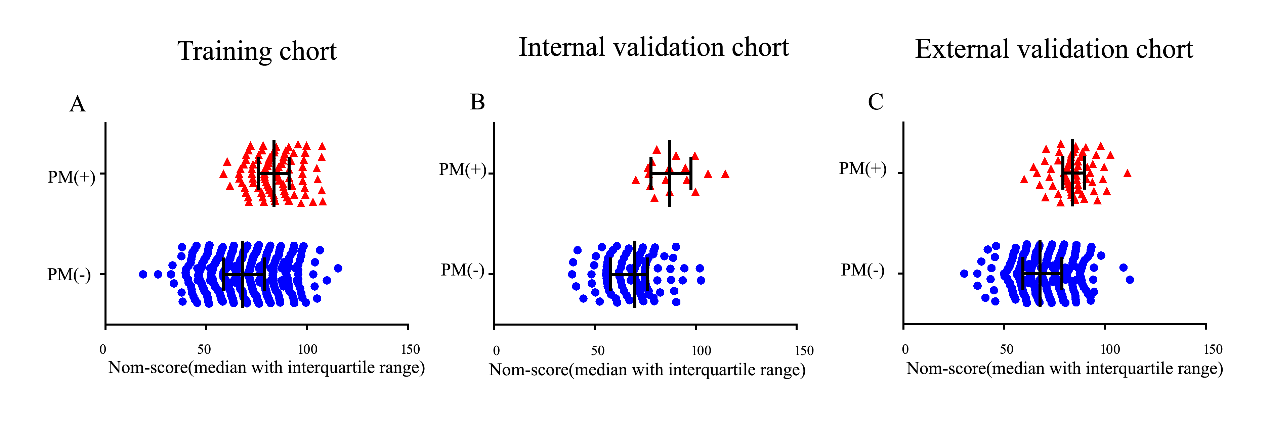


**Figure S7: Distribution of Nomogram scores according to the PM status.**

(A), (B), (C): The values of the Nomogram scores (Nom-scores) of each patient and the median values with interquartile range of Nom-scores. PM, peritoneal metastasis status.

| **Table S1.** Descriptive statistics for gastric cancer in the training cohort. | | | | |
| --- | --- | --- | --- | --- |
| **Variables** | **Training Cohort (N=562)** | | | ***P*** |
|  | Number | PM(-)(472) | PM(+)(90) |  |
| **Age (years)** |  |  | | 0.155 |
| ≥60 | 238 | 206(43.6) | 32(35.6) |  |
| < 60 | 324 | 266(56.4) | 58(64.4) |  |
| **Gender** |  |  |  | 0.249 |
| Male | 385 | 328(69.5) | 57(63.3) |  |
| Female | 177 | 144(30.5) | 33(36.7) |  |
| **Size** |  |  |  | 0.245 |
| ≥4cm | 409 | 339(71.8) | 70(77.8) |  |
| < 4cm | 153 | 133(28.2) | 20(22.2) |  |
| **Differentiation** |  |  |  | 0.175 |
| Well or Moderate | 75 | 67(14.2) | 8(8.9) |  |
| Poor or undifferentiated | 487 | 405(85.8) | 82(91.1) |  |
| **Lauren type** |  |  |  | 0.583 |
| intestinal | 157 | 134(28.4) | 23(25.6) |  |
| mixed and diffuse | 405 | 338(71.6) | 67(74.4) |  |
| **Location** |  |  |  | 0.005 |
| Cardia | 218 | 197(41.7) | 21(23.3) |  |
| Body | 113 | 93(19.7) | 20(22.2) |  |
| Antrum | 183 | 147(31.0) | 36(40.0) |  |
| Whole | 48 | 35(7.4) | 13(14.4) |  |
| **CEA** |  |  |  | 0.319 |
| elevated | 139 | 113(23.9) | 26(28.9) |  |
| normal | 423 | 359(76.1) | 64(71.1) |  |
| **CA19-9** |  |  |  | 0.254 |
| elevated | 130 | 105(22.2) | 25(27.8) |  |
| normal | 432 | 367(77.8) | 65(72.2) |  |
| **cT** |  |  |  | 0.002 |
| T3 | 99 | 92(19.5) | 7(7.8) |  |
| T4a | 337 | 285(60.4) | 52(57.8) |  |
| T4b | 126 | 95(20.1) | 31(34.4) |  |
| **cN** |  |  |  | <0.0001 |
| N0 | 159 | 148(31.4) | 11(12.2) |  |
| N1 | 131 | 113(23.9) | 18(20) |  |
| N2 | 125 | 99(21) | 26(28.9) |  |
| N3 | 147 | 112(23.7) | 35(38.9) |  |
| **Rad-score, mean(95%CI)** | 562 | -0.138(-0.277, 0.0001) | 0.123(0.097,0.150) | <0.0001 |

PM(+), PM-positive status; PM(-), PM-negative status

| **Table S2.** Descriptive statistics for gastric cancer in the internal validation cohorts. | | | | |
| --- | --- | --- | --- | --- |
| **Variables** | **Internal Validation Cohort (N=106)** | | | ***P*** |
|  | Number | PM(-)(89) | PM(+)(17) |  |
| **Age (years)** |  |  | | 0.739 |
| ≥60 | 46 | 38(42.7) | 8(47.1) |  |
| < 60 | 60 | 51(57.3) | 9(52.9) |  |
| **Gender** |  |  |  | 0.912 |
| Male | 76 | 64(71.9) | 12(70.6) |  |
| Female | 30 | 25(28.1) | 5(29.4) |  |
| **Size** |  |  |  | 0.419 |
| ≥4cm | 79 | 65(73) | 24(27) |  |
| < 4cm | 27 | 24(27) | 3(17.6) |  |
| **Differentiation** |  |  |  | 0.807 |
| Well or Moderate | 21 | 18(20.2) | 3(17.6) |  |
| Poor or undifferentiated | 85 | 71(79.8) | 14(82.4) |  |
| **Lauren type** |  |  |  | 0.008 |
| intestinal | 36 | 35(39.3) | 1(5.9) |  |
| mixed and diffuse | 70 | 54(60.7) | 16(94.1) |  |
| **Location** |  |  |  | 0.108 |
| Cardia | 32 | 31(34.8) | 1(5.9) |  |
| Body | 31 | 24(27.0) | 7(41.2) |  |
| Antrum | 36 | 29(32.6) | 7(41.2) |  |
| Whole | 7 | 5(5.6) | 2(11.8) |  |
| **CEA** |  |  |  | 0.485 |
| elevated | 30 | 24(27.0) | 6(35.3) |  |
| normal | 76 | 65(73.0) | 11(64.7) |  |
| **CA19-9** |  |  |  | 0.178 |
| elevated | 19 | 14(15.7) | 5(29.4) |  |
| normal | 87 | 75(84.3) | 12(70.6) |  |
| **cT** |  |  |  | 0.013 |
| T3 | 27 | 25(28.1) | 2(11.8) |  |
| T4a | 48 | 43(48.3) | 5(29.4) |  |
| T4b | 31 | 21(23.6) | 10(58.8) |  |
| **cN** |  |  |  | 0.013 |
| N0 | 23 | 23(25.8) | 0(0.0) |  |
| N1 | 18 | 17(19.1) | 1(5.9) |  |
| N2 | 22 | 15(16.9) | 7(41.2) |  |
| N3 | 43 | 34(38.2) | 9(52.9) |  |
| **Rad-score, mean(95%CI)** | 106 | -0.025(-0.053,0.004) | 0.139(0.063,0.214) | <0.0001 |

PM(+), PM-positive status; PM(-), PM-negative status

| **Table S3.** Descriptive statistics for gastric cancer in the external validation cohorts. | | | | |
| --- | --- | --- | --- | --- |
| **Variables** | **External Validation Cohort (N=287)** | | | ***P*** |
|  | Number | PM(-)(225) | PM(+)(62) |  |
| **Age (years)** |  |  | | 0.385 |
| ≥60 | 125 | 101(44.9) | 24(38.7) |  |
| < 60 | 162 | 124(55.1) | 38(61.3) |  |
| **Gender** |  |  |  | 0.928 |
| Male | 207 | 162(72.0) | 45(72.6) |  |
| Female | 80 | 63(28.0) | 17(27.4) |  |
| **Size** |  |  |  | 0.045 |
| ≥4cm | 196 | 147(65.3) | 49(79.0) |  |
| < 4cm | 91 | 78(34.7) | 13(21.0) |  |
| **Differentiation** |  |  |  | 0.734 |
| Well or Moderate | 60 | 48(21.3) | 12(19.4) |  |
| Poor or undifferentiated | 227 | 177(78.7) | 50(80.6) |  |
| **Lauren type** |  |  |  | 0.306 |
| intestinal | 100 | 75(33.3) | 25(40.3) |  |
| mixed and diffuse | 187 | 150(66.7) | 37(59.7) |  |
| **Location** |  |  |  | 0.724 |
| Cardia | 59 | 46(20.4) | 13(21.0) |  |
| Body | 48 | 37(16.4) | 11(17.7) |  |
| Antrum | 140 | 113(50.2) | 27(43.5) |  |
| Whole | 40 | 29(12.9) | 11(17.7) |  |
| **CEA** |  |  |  | 0.406 |
| elevated | 50 | 37(16.4) | 13(21.0) |  |
| normal | 237 | 188(83.6) | 49(79.0) |  |
| **CA19-9** |  |  |  | 0.891 |
| elevated | 62 | 49(21.8) | 13(21.0) |  |
| normal | 225 | 176(78.2) | 49(79.0) |  |
| **cT** |  |  |  | <0.0001 |
| T3 | 34 | 34(15.1) | 0(0.0) |  |
| T4a | 232 | 183(81.3) | 49(79.0) |  |
| T4b | 21 | 8(3.6) | 13(21.0) |  |
| **cN** |  |  |  | <0.0001 |
| N0 | 69 | 66(29.3) | 3(4.8) |  |
| N1 | 58 | 51(22.7) | 7(11.3) |  |
| N2 | 69 | 42(18.7) | 27(43.5) |  |
| N3 | 91 | 66(29.3) | 25(40.3) |  |
| **Rad-score, mean(95%CI)** | 287 | -0.011(-0.031,0.008) | 0.109(0.081,0.138) | <0.0001 |

PM(+), PM-positive status; PM(-), PM-negative status

| **Table S4.** Stratified analysis of the association between the radiomics signature and Peritoneal metastasis in the training cohort. | | | | |
| --- | --- | --- | --- | --- |
| **Variables** | **Rad-score (Training Cohort, N=562)** | | | ***P*** |
|  | Number | PM(-)(472) | PM(+)(90) |  |
| **Age (years)** |  |  | |  |
| ≥60 | 238 | -0.018(-0.039, 0.004) | 0.116(0.067, 0.165) | <0.0001 |
| < 60 | 324 | -0.011(-0.030, 0.008) | 0.127(0.095, 0.160) | <0.0001 |
| **Gender** |  |  |  |  |
| Male | 385 | -0.014(-0.031, 0.003) | 0.135(0.099, 0.170) | <0.0001 |
| Female | 177 | -0.014(-0.039, 0.011) | 0.104(0.063, 0.146) | <0.0001 |
| **Size** |  |  |  |  |
| ≥4cm | 409 | -0.002(-0.018, 0.015) | 0.129(0.097, 0.161) | <0.0001 |
| < 4cm | 153 | -0.045(-0.070, -0.020) | 0.103(0.054, 0.152) | <0.0001 |
| **Lauren type** |  |  |  |  |
| intestinal | 157 | -0.038(-0.062, 0.014) | 0.159(0.099, 0.218) | <0.0001 |
| mixed and diffuse | 405 | -0.004(-0.021, 0.125) | 0.111(0.081, 0.141) | <0.0001 |
| **Differentiation** |  |  |  |  |
| Well or | 75 | -0.035(-0.069, -0.001) | 0.219(0.132, 0.306) | <0.0001 |
| Moderate |  |  |  |  |
| Poor or | 487 | -0.010(-0.026, 0.005) | 0.114(0.086, 0.142) | <0.0001 |
| undifferentiated |  |  |  |  |
| **Location** |  |  |  |  |
| Cardia | 218 | -0.013(-0.032, 0.006) | 0.185(0.125, 0.246) | <0.0001 |
| Body | 113 | -0.034(-0.068, -0.001) | 0.129(0.074, 0.183) | <0.0001 |
| Antrum | 183 | -0.017(-0.045, 0.010) | 0.102(0.063, 0.141) | <0.0001 |
| Whole | 48 | 0.049(-0.002, 0.101) | 0.075(-0.009, 0.160) | 0.582 |
| **CEA** |  |  |  |  |
| elevated | 139 | -0.012(-0.037, 0.137) | 0.134(0.076, 0.191) | <0.0001 |
| normal | 423 | -0.014(-0.031, 0.002) | 0.119(0.089, 0.150) | <0.0001 |
| **CA19-9** |  |  |  |  |
| elevated | 130 | -0.003(-0.030, 0.024) | 0.149(0.099, 0.198) | <0.0001 |
| normal | 432 | -0.017(-0.033, -0.001) | 0.114(0.082, 0.146) | <0.0001 |
| **cT stage** |  |  |  |  |
| T3 | 99 | -0.050(-0.078, -0.022) | 0.142(0.015, 0.269) | 0.009 |
| T4a | 337 | -0.013(-0.032, 0.005) | 0.141(0.104, 0.178) | <0.0001 |
| T4b | 126 | 0.019(-0.011, 0.049) | 0.090(0.048, 0.132) | 0.007 |
| **cN stage** |  |  |  |  |
| N0 | 159 | -0.018(-0.041, 0.006) | 0.100(0.016, 0.183) | 0.011 |
| N1 | 131 | -0.020(-0.049, 0.009) | 0.122(0.059, 0.185) | <0.0001 |
| N2 | 125 | -0.013(-0.042, 0.015) | 0.138(0.083, 0.193) | <0.0001 |
| N3 | 147 | -0.003(-0.035, 0.290) | 0.121(0.078, 0.165) | <0.0001 |

PM(+), PM-positive status; PM(-), PM-negative status

| **Table S5.** Stratified analysis of the association between the radiomics signature and Peritoneal metastasis in the internal validation cohort. | | | | |
| --- | --- | --- | --- | --- |
| **Variables** | **Rad-score (Internal Validation Cohort, N=106)** | | | ***P*** |
|  | Number | PM(-)(89) | PM(+)(17) |  |
| **Age (years)** |  |  | |  |
| ≥60 | 46 | -0.003(-0.045, 0.039) | 0.128(0.024, 0.233) | 0.022 |
| < 60 | 60 | -0.041(-0.081, -0.001) | 0.148(0.016, 0.280) | 0.011 |
| **Gender** |  |  |  |  |
| Male | 76 | -0.028(-0.063, 0.008) | 0.146(0.040, 0.251) | <0.0001 |
| Female | 30 | -0.017(-0.067, 0.033) | 0.122(-0.004, 0.248) | 0.024 |
| **Size** |  |  |  |  |
| ≥4cm | 79 | -0.001(-0.036, 0.034) | 0.156(0.068, 0.243) | <0.0001 |
| < 4cm | 27 | -0.089(-0.129, -0.049) | 0.059(-0.221, 0.339) | 0.019 |
| **Lauren type** |  |  |  |  |
| intestinal | 36 | -0.030(-0.063, 0.003) | 0.135 | 0.098 |
| mixed and diffuse | 70 | -0.021(-0.064, 0.022) | 0.139(0.058,0.220) | 0.001 |
| **Differentiation** |  |  |  |  |
| Well or | 21 | -0.013(-0.063, 0.038) | 0.136(-0.072, 0.343) | 0.027 |
| Moderate |  |  |  |  |
| Poor or | 85 | -0.028(-0.062, 0.006) | 0.139(0.047, 0.232) | <0.0001 |
| undifferentiated |  |  |  |  |
| **Location** |  |  |  |  |
| Cardia | 32 | -0.042(-0.087, 0.002) | 0.152 | 0.124 |
| Body | 31 | -0.015(-0.073, 0.043) | 0.142(0.045, 0.240) | 0.007 |
| Antrum | 36 | -0.046(-0.098, 0.006) | 0.128(-0.059, 0.314) | 0.01 |
| Whole | 7 | 0.158(0.043, 0.272) | 0.157(-1.481, 1.795) | 0.998 |
| **CEA** |  |  |  |  |
| elevated | 30 | -0.018(-0.076, 0.041) | 0.133(-0.032, 0.297) | 0.028 |
| normal | 76 | -0.028(-0.061, 0.006) | 0.142(0.042, 0.242) | <0.0001 |
| **CA19-9** |  |  |  |  |
| elevated | 19 | 0.012(-0.108, 0.131) | 0.234(0.023, 0.445) | 0.043 |
| normal | 87 | -0.032(-0.059, -0.004) | 0.099(0.021, 0.177) | 0.004 |
| **cT stage** |  |  |  |  |
| T3 | 27 | -0.06(-0.104, -0.017) | 0.144(0.035, 0.253) | 0.013 |
| T4a | 48 | -0.002(-0.050, 0.046) | 0.131(0.022, 0.240) | 0.067 |
| T4b | 31 | -0.029(-0.085, 0.026) | 0.141(0.008, 0.275) | 0.005 |
| **cN stage** |  |  |  |  |
| N0 | 23 | -0.034(-0.095, 0.026) | / | / |
| N1 | 18 | 0.017(-0.062, 0.096) | 0.045 | 0.86 |
| N2 | 22 | -0.038(-0.115, 0.039) | 0.163(-0.018, 0.345) | 0.012 |
| N3 | 43 | -0.034(-0.077, 0.010) | 0.130(0.043, 0.216) | 0.001 |

PM(+), PM-positive status; PM(-), PM-negative status

| **Table S**6. Stratified analysis of the association between the radiomics signature and Peritoneal metastasis in the external validation cohort. | | | | |
| --- | --- | --- | --- | --- |
| **Variables** | **Rad-score (External Validation Cohort, N=287)** | | | ***P*** |
|  | Number | PM(-)(225) | PM(+)(62) |  |
| **Age (years)** |  |  | |  |
| ≥60 | 125 | -0.011(-0.037, 0.014) | 0.126(0.075, 0.176) | <0.0001 |
| < 60 | 162 | -0.012(-0.041, 0.018) | 0.099(0.064, 0.135) | <0.0001 |
| **Gender** |  |  |  |  |
| Male | 207 | -0.005(-0.028, 0.179) | 0.112(0.078, 0.145) | <0.0001 |
| Female | 80 | -0.027(-0.065, 0.010) | 0.103(0.044, 0.163) | 0.001 |
| **Size** |  |  |  |  |
| ≥4cm | 196 | -0.011(-0.035, 0.013) | 0.109(0.077, 0.141) | <0.0001 |
| < 4cm | 91 | -0.013(-0.047, 0.022) | 0.110(0.039, 0.182) | 0.004 |
| **Lauren type** |  |  |  |  |
| intestinal | 100 | -0.036(-0.066, -0.005) | 0.114(0.069, 0.160) | <0.0001 |
| mixed and diffuse | 187 | 0.001(-0.024, 0.026) | 0.106(0.067, 0.145) | <0.0001 |
| **Differentiation** |  |  |  |  |
| Well or | 60 | -0.019(-0.060, 0.023) | 0.056(-0.012, 0.125) | 0.058 |
| Moderate |  |  |  |  |
| Poor or | 227 | -0.009(-0.032, 0.013) | 0.122(0.091, 0.154) | <0.0001 |
| undifferentiated |  |  |  |  |
| **Location** |  |  |  |  |
| Cardia | 59 | -0.015(-0.051, 0.022) | 0.164(0.078, 0.249) | <0.0001 |
| Body | 48 | -0.023(-0.072, 0.026) | 0.134(0.068, 0.199) | 0.002 |
| Antrum | 140 | -0.024(-0.050, 0.003) | 0.107(0.068, 0.145) | <0.0001 |
| Whole | 40 | 0.056(-0.019, 0.131) | 0.028(-0.031, 0.086) | 0.528 |
| **CEA** |  |  |  |  |
| elevated | 40 | -0.018(-0.070, 0.035) | 0.140(0.070, 0.210) | 0.002 |
| normal | 237 | -0.010(-0.031, 0.011) | 0.101(0.069, 0.133) | <0.0001 |
| **CA19-9** |  |  |  |  |
| elevated | 62 | -0.005(-0.052,0.042) | 0.103(0.028, 0.178) | 0.016 |
| normal | 225 | -0.013(-0.035,0.008) | 0.111(0.079, 0.143) | <0.0001 |
| **cT stage** |  |  |  |  |
| T3 | 34 | -0.035(-0.078, 0.008) | / | / |
| T4a | 232 | -0.006(-0.028, 0.016) | 0.106(0.073, 0.140) | <0.0001 |
| T4b | 21 | -0.039(-0.168, 0.090) | 0.120(0.058, 0.182) | 0.025 |
| **cN stage** |  |  |  |  |
| N0 | 69 | 0.004(-0.031, 0.038) | 0.145(-0.278, 0.567) | 0.286 |
| N1 | 58 | -0.011(-0.051, 0.030) | 0.107(0.020, 0.193) | 0.016 |
| N2 | 69 | 0.018(-0.034, 0.070) | 0.108(0.062, 0.153) | 0.011 |
| N3 | 91 | -0.046(-0.081, -0.010) | 0.108(0.061, 0.155) | <0.0001 |

PM(+), PM-positive status; PM(-), PM-negative status

| **Table S7.** Univariate association of Rad-score, clinicopathological characteristics with Peritoneal metastasis in the training, internal and external validation cohorts. | | | | | | | |
| --- | --- | --- | --- | --- | --- | --- | --- |
| **Variables** | **PM(+) vs. PM(-)** | | | | | | |
|  | OR (95%CI) | *P* | OR (95%CI) | *P* | OR (95%CI) | | *P* |
|  | **Training cohort** | | **Internal validation cohort** | | | **External validation cohort** | |
|  |  | |  | | |  | |
| **Rad-score*** | **6.670(3.670-12.336)** | **<0.0001** | **9.187(1.983-42.573)** | **0.005** | **6.851(3.225-14.558)** | | **<0.0001** |
| Age(years) (≥60 vs. <60) | 0.712(0.446-1.138) | 0.156 | 1.193(0.421-3.378) | 0.74 | 0.775(0.436-1.377) | | 0.386 |
| Gender (male vs. female) | 0.758(0.473-1.215) | 0.25 | 0.938(0.3-2.935) | 0.912 | 1.029(0.549-1.931) | | 0.928 |
| **Location** |  |  |  |  |  | |  |
| Cardia | 0.287(0.132-0.626) | 0.002 | 0.081(0.006-1.064) | 0.056 | 0.745(0.295-1.884) | | 0.534 |
| Body | 0.579(0.260-1.287) | 0.18 | 0.729(0.115-4.607) | 0.737 | 0.784(0.298-2.061) | | 0.621 |
| Antrum | 0.659(0.317-1.373) | 0.266 | 0.603(0.096-3.784) | 0.59 | 0.630(0.280-1.418) | | 0.264 |
| Whole | Reference |  | Reference |  | Reference | |  |
| Tumor size  (≥4cm vs. <4cm) | 1.373(0.804-2.347) | 0.246 | 1.723(0.455-6.528) | 0.423 | 2.000(1.023-3.910) | | 0.043 |
| Differentiation  (Poor or undifferentiation vs. Well or Moderate) | 1.696(0.785-3.665) | 0.179 | 1.183(0.307-4.564) | 0.807 | 1.130(0.558-2.290) | | 0.735 |
| CEA (elevated vs. Normal) | 1.291(0.781-2.133) | 0.32 | 1.477(0.492-4.435) | 0.487 | 1.348(0.666-2.730) | | 0.407 |
| CA19-9(elevated vs. Normal) | 1.344(0.807-2.238) | 0.255 | 2.232(0.680-7.331) | 0.186 | 0.953(0.479-1.897) | | 0.891 |
| **cT stage** |  |  |  |  |  | |  |
| T3 | 0.233(0.098-0.556) | 0.001 | 0.168(0.033-0.853) | 0.031 | / | | 0.997 |
| T4a | 0.559(0.339-0.923) | 0.023 | 0.244(0.074-0.806) | 0.021 | 0.165(0.065-0.420) | | <0.0001 |
| T4b | Reference |  | Reference |  | Reference | |  |
| **cN stage** |  |  |  |  |  | |  |
| N0 | 0.238(0.116-0.489) | <0.0001 | / | 0.998 | 0.120(0.035-0.417) | | 0.001 |
| N1 | 0.510(0.273-0.953) | 0.035 | 0.222(0.026-1.901) | 0.17 | 0.362(0.145-0.904) | | 0.03 |
| N2 | 0.840(0.473-1.493) | 0.553 | 1.763(0.553-5.622) | 0.338 | 1.697(0.871-3.308) | | 0.12 |
| N3 | Reference |  | Reference |  | Reference | |  |
| *, Rad-score is a continuous value with a scale of 1; PM(+), PM-positive status; PM(-), PM-negative status; OR, odds ratio CI, confidence interval. | | | | | | | |

| **Table S8.** The diagnostic performance of Rad-score and Nomogram in predicting gastric cancer peritoneal metastasis in the training cohort, internal validation cohort and external validation cohort. | | | | | | | | | |
| --- | --- | --- | --- | --- | --- | --- | --- | --- | --- |
|  | Cutoff | AUC | Sen | Spe | Acc | +LR | -LR | PPV | NPV |
| Training cohort |  |  |  |  |  |  |  |  |  |
| Rad-score | 0.0002 | 0.751 | 0.844 | 0.530 | 0.58 | 1.80 | 0.29 | 0.255 | 0.95 |
| Nomogram | 77.431 | 0.792 | 0.733 | 0.723 | 0.724 | 2.64 | 0.37 | 0.335 | 0.934 |
|  |  |  |  |  |  |  |  |  |  |
| Internal validation cohort | |  |  |  |  |  |  |  |  |
| Rad-score | 0.0002 | 0.802 | 0.882 | 0.562 | 0.613 | 2.01 | 0.21 | 0.278 | 0.962 |
| Nomogram | 77.431 | 0.87 | 0.765 | 0.787 | 0.783 | 3.58 | 0.3 | 0.406 | 0.946 |
|  |  |  |  |  |  |  |  |  |  |
| External validation cohort | |  |  |  |  |  |  |  |  |
| Rad-score | 0.0002 | 0.745 | 0.855 | 0.538 | 0.606 | 1.85 | 0.27 | 0.338 | 0.931 |
| Nomogram | 77.431 | 0.815 | 0.79 | 0.729 | 0.742 | 2.92 | 0.29 | 0.445 | 0.927 |
| AUC, area under the receiver operating characteristic curve; Sen, sensitivity; Spe, specificity; Acc, accuracy; +LR, positive likelihood ratio; −LR, negative likelihood ratio; PPV, positive predictive value; NPV, negative predictive value | | | | | | | | | |
